# Progress in Borneol Intervention for Ischemic Stroke: A Systematic Review

**DOI:** 10.3389/fphar.2021.606682

**Published:** 2021-05-04

**Authors:** Yong Li, Mihong Ren, Jiajun Wang, Rong Ma, Hai Chen, Qian Xie, Hongyan Li, Jinxiu Li, Jian Wang

**Affiliations:** College of Pharmacy, Chengdu University of Traditional Chinese Medicine, Chengdu, China

**Keywords:** L-borneol, D-borneol, DL-borneol, ischemic stroke, cascade reaction, neuroprotection

## Abstract

**Background:** Borneol is a terpene and bicyclic organic compound that can be extracted from plants or chemically synthesized. As an important component of proprietary Chinese medicine for the treatment of stroke, its neuroprotective effects have been confirmed in many experiments. Unfortunately, there is no systematic review of these studies. This study aimed to systematically examine the neuroprotective effects of borneol in the cascade reaction of experimental ischemic stroke at different periods.

**Methods:** Articles on animal experiments and cell-based research on the actions of borneol against ischemic stroke in the past 20°years were collected from Google Scholar, Web of Science, PubMed, ScienceDirect, China National Knowledge Infrastructure (CNKI), and other biomedical databases. Meta-analysis was performed on key indicators *in vivo* experiments. After sorting the articles, we focused on the neuroprotective effects and mechanism of action of borneol at different stages of cerebral ischemia.

**Results:** Borneol is effective in the prevention and treatment of nerve injury in ischemic stroke. Its mechanisms of action include improvement of cerebral blood flow, inhibition of neuronal excitotoxicity, blocking of Ca^2+^ overload, and resistance to reactive oxygen species injury in the acute ischemic stage. In the subacute ischemic stage, borneol may antagonize blood-brain barrier injury, intervene in inflammatory reactions, and prevent neuron excessive death. In the late stage, borneol promotes neurogenesis and angiogenesis in the treatment of ischemic stroke.

**Conclusion:** Borneol prevents neuronal injury after cerebral ischemia via multiple action mechanisms, and it can mobilize endogenous nutritional factors to hasten repair and regeneration of brain tissue. Because the neuroprotective effects of borneol are mediated by various therapeutic factors, deficiency caused by a single-target drug is avoided. Besides, borneol promotes other drugs to pass through the blood-brain barrier to exert synergistic therapeutic effects.

## Introduction

**FIGURE 1:**
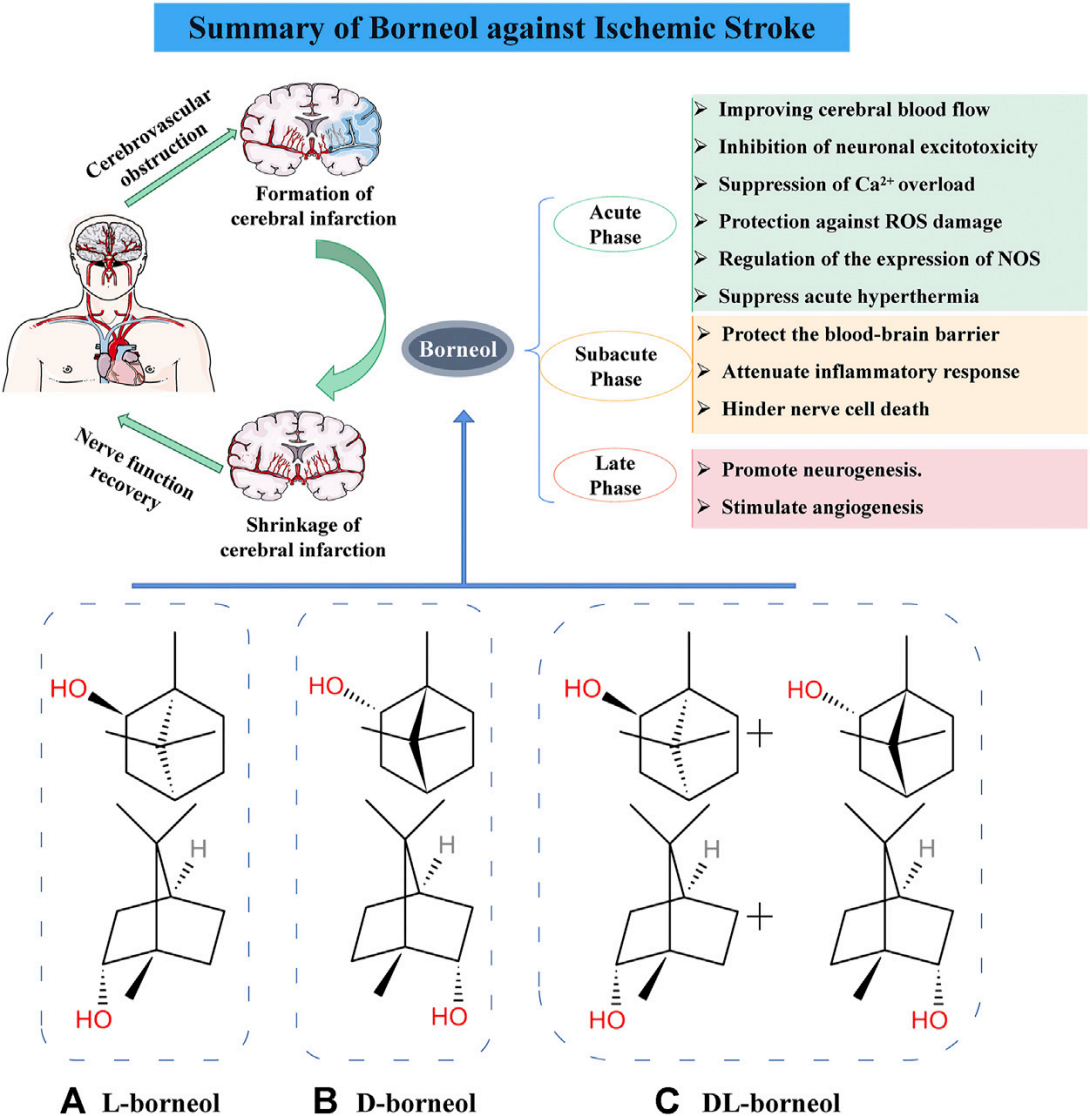
Mechanism of borneol against cerebral ischemia injury and chemical structure of three borneols. **(A)** Levorotatory borneol (endo-(1 S)-1,7,7-trimethyl-bicyclo [2.2.1] heptan-2-ol, (–)-borneol), which is extracted from fresh leaves of Blumea balsamifera (L.) DC. **(B)** Dextrorotatory borneol (endo-(1R)-1,7,7-trimethyl-bicyclo [2.2.1] heptan-2-ol, (+)-borneol), which is extracted from fresh branches and leaves of Cinnamomum camphora (L.) Presl. **(C)** Synthetic borneol (DL-borneol) is an optically inactive (±) borneol that is mainly a mixture of (±) borneol and is obtained via the chemical transformation of camphor and turpentine oil.

Borneol is a bicyclic terpenoid with strong fat solubility (Lv et al., 2012). The 2020 edition of Chinese Pharmacopoeia contains three commercial borneol products: D-borneol (Chinese name “Tianranbingpian”), L-borneol (Chinese name “Aipian”), and Synthetic borneol (Chinese name “Bingpian”). D-borneol are extracted from fresh branches and leaves of *Cinnamo* *mum camphora* (L.) Presl.or *Dryobalanops aromatica Gaertn.f* (Yang et al., 2018; Wang and Wang, 2018)*.* L-borneol is processed from the sublimation of *Blumea balsamifera (L.) DC* (Islam et al., 2010)*.* Synthetic borneol is a racemic borneol (A mixture with D-borneol and L-borneol as the main components) prepared by chemical synthesis of turpentine and camphor (Chinese Pharmacopoeia Committee, 2020) (Figure 1). Borneol has been used in China for more than 1600 years (Li et al., 2013). In Traditional Chinese Medicine (TCM) theory, borneol is a upper ushering drug that guides herbs to their target organs, especially in the upper part of the body, including the brain (Chen et al., 2019). In addition, borneol is suitable for the treatment of mental diseases accompanied by signs of heat syndrome because the herbal medicine has the property of being “cold” (Zou et al., 2017; Wang and Wang, 2018). Several proprietary Chinese medicine preparations such as Angong Niuhuang pills and Xingnaojing injection contain borneol and are widely used in the clinical treatment of stroke (Guo et al., 2014; Zhang et al., 2019).

Cerebral stroke is a disorder of cerebral blood circulation of the central nervous system and may either be ischemic or hemorrhagic. Ischemic stroke accounts for approximately 80% of stroke cases (Fan et al., 2017). Ischemic stroke is accompanied by extremely complex physiological and pathological processes. The acute stage of ischemic stroke is characterized by glutamate excitotoxicity, intracellular Ca^2+^ overload, oxidative stress, and production of free radicals. The subacute stage is characterized by apoptosis and necrosis, blood-brain barrier damage, brain edema, and an inflammatory response. The late stage of ischemic stroke is characterized by reactive astrocyte proliferation, glial scar formation, angiogenesis, and neurogenesis (Steliga et al., 2020). These processes occur at different time points, overlap with each other, and eventually lead to brain injury and repair after ischemia.

In the study of brain diseases, the regulatory effect of borneol on blood-brain barrier has always been the focus of relevant practitioners. In fact, there are also abundant studies have shown that borneol has a neuroprotective effect on cerebral ischemic. Unfortunately, the neuroprotective mechanism of borneol is complex and involves many aspects, and only few reviews have focused on the protective mechanism of borneol at different periods of cerebral ischemia. Therefore, we take the review of borneol intervention in cerebral ischemia as the main content of the article, and supplement the meta-analysis of key indicators *in vivo* studies, hoping to provide some valuable references for borneol’s experimental research and clinical application.

## Methods

### Search Strategy

A comprehensive search strategy was conducted in several databases, including Google Scholar, Web of Science, ScienceDirect, PubMed, and CNKI from their inceptions to March 2021. For data mining, the following words were used in the databases mentioned above: “borneol” or “D-borneol” or “L-borneol” or “synthetic borneol” and “cerebral ischemic” or “ischemic stroke” or “neuroprotection.” In almost all cases, the original articles or abstracts were obtained and the relevant data was extracted.

### Eligibility and Exclusion Criteria

**FIGURE 2:**
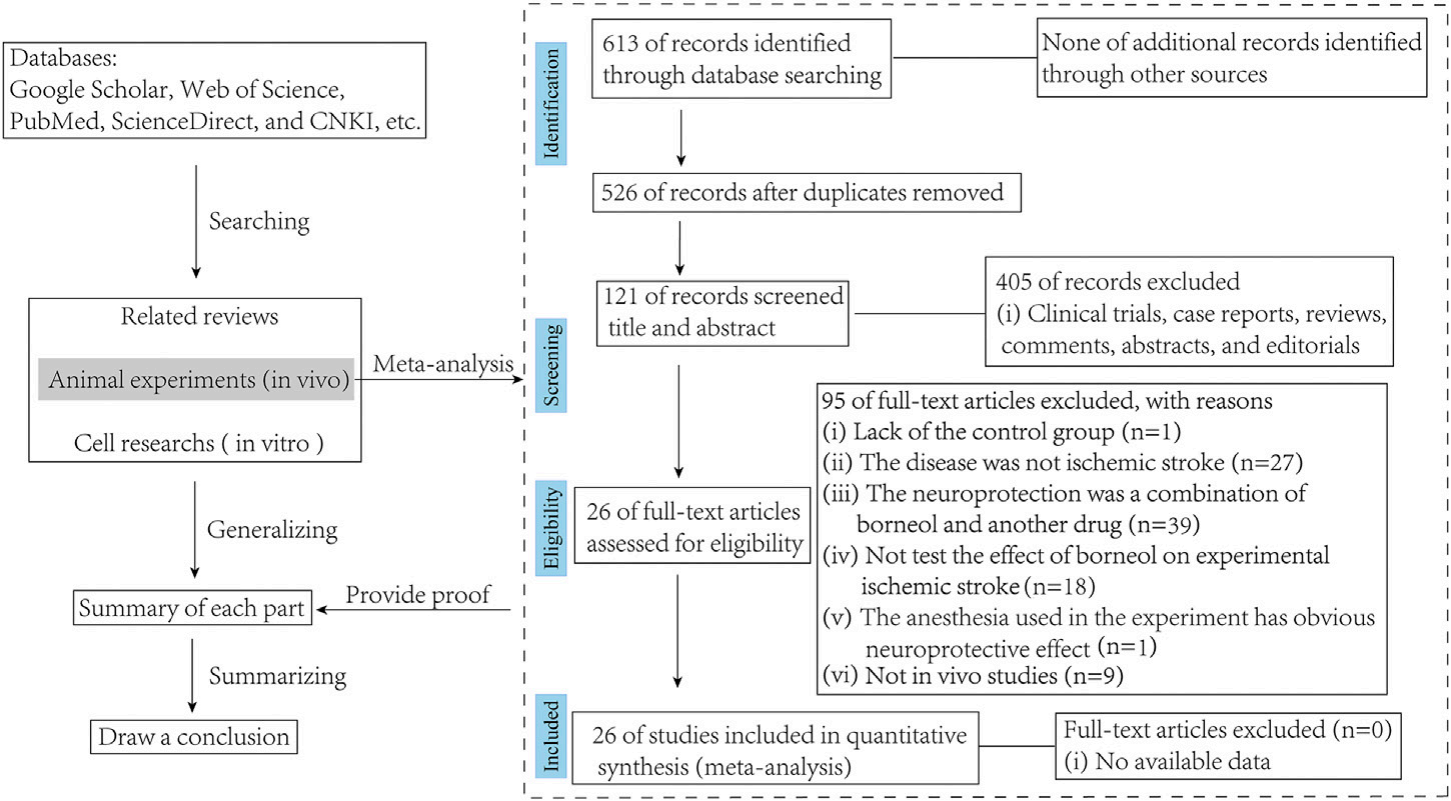
Flow diagram of the search process.

Both animal experiments (*in vivo*) and cell studies (*in vitro*) on borneol intervention in ischemic stroke have been included in the review. But all studies accepted for meta-analysis should be met the following eligibility criteria: 1) The drugs used must be borneol, whether D-borneol, L-borneol, or synthetic borneol, it should be noted that the literature on combination of drugs, which sets borneol alone as a group to compare with the model group, can also be included in this study; 2) The animal model used must be a cerebral ischemia model, whether permanent or cerebral ischemia-reperfusion; and 3) the control group receiving vehicle or no adjunct intervention was included in the studies. Exclusion criteria were as follows: 1) the study was a case report, clinical trial, review, or *in vitro* study; 2) lack of the control group; 3) the targeting disease was not ischemic stroke; 4) the intervention was a combination of borneol and another agent with potential effect on ischemic stroke; 5) the anesthesia used in the experiment has obvious neuroprotective effect (Figure 2).

### Statistical Analysis

To evaluate the effect of borneol on cerebral ischemia in animal experiments, Revman version 5.3 was performed for statistical analysis. If statistical heterogeneity was found (*p* < 0.1, *I* ^2^ > 50%), a model of random effect (RE) was applied to evaluate pooled effect with 95% CI; and if no statistical evidence of heterogeneity existed (*p* ≥ 0.1, *I* ^2^ ≤ 50%), a model of fixed effect (FE) was set with 95% CI. If the outcomes were applied at the same scale, the weighted mean difference (WMD) was calculated as a summary statistic; and if the same results were measured in different ways, the standardized mean differences (SMD) were used. Heterogeneity was assessed by standard chi-square test and *I* ^2^ statistics. A probability value less than 0.05 was considered statistically significant. To minimize bias and human error, the meta-analysis was performed by 2 independent reviewers and disagreements reconciled by a third independent reviewer.

## Results

### Mechanism of Borneol Intervention in the Acute Stage of Ischemic Stroke

Decreased cerebral blood flow in the acute phase of ischemic stroke (within 24 h of human cerebral ischemia) causes a decrease in oxygen and metabolic substrates to neurons. Sbusequently, The lack of oxygen interrupts oxidative phosphorylation by the mitochondria and drastically reduces cellular ATP production. Inhibition of the Na^+^/K^+^-ATPase function causes a profound loss of ionic gradients and depolarization of regulated neurons, which leads to excessive release of excitatory amino acids-particularly glutamate-to the extracellular compartment. The presence of excessive amounts of free glutamate into the synapses and extrasynaptic sites can lead eventually to neuronal death. Excitotoxicity leads to a number of deleterious consequences, including impairment of cellular calcium homeostasis, generation of free radicals and oxidative stress, mitochondrial damage, and activation of transcription factors. All these mechanisms’ acting synergy can cause acute neuron death by apoptosis (Rama and Garcia, 2016). Borneol participates in multiple physiological and pathological processes in the acute phase of ischemic stroke and impedes disease progression by antagonizing the damage in the initial stage of ischemic stroke.

#### Improving Cerebral Blood Flow

Disordered energy metabolism is required in the development of cerebral ischemia. Stenosis and occlusion of blood vessels lead to the interruption of local blood flow and alters blood circulation. Cerebral infarction occurs when the supply of nutrients provided by local blood circulation does not meet the energy metabolism needs of brain cells beyond a certain time limit. Therefore, timely improvement of the blood supply to the brain is the primary goal of the clinical treatment of ischemic stroke. Borneol has the pharmacological effects of relaxing blood vessels, reducing blood pressure and cerebral vascular resistance, inhibiting thrombosis, and promoting thrombolysis, thereby improving cerebral blood flow after cerebral ischemia.

##### Regulation of Vascular Neuropeptide

**FIGURE 3:**
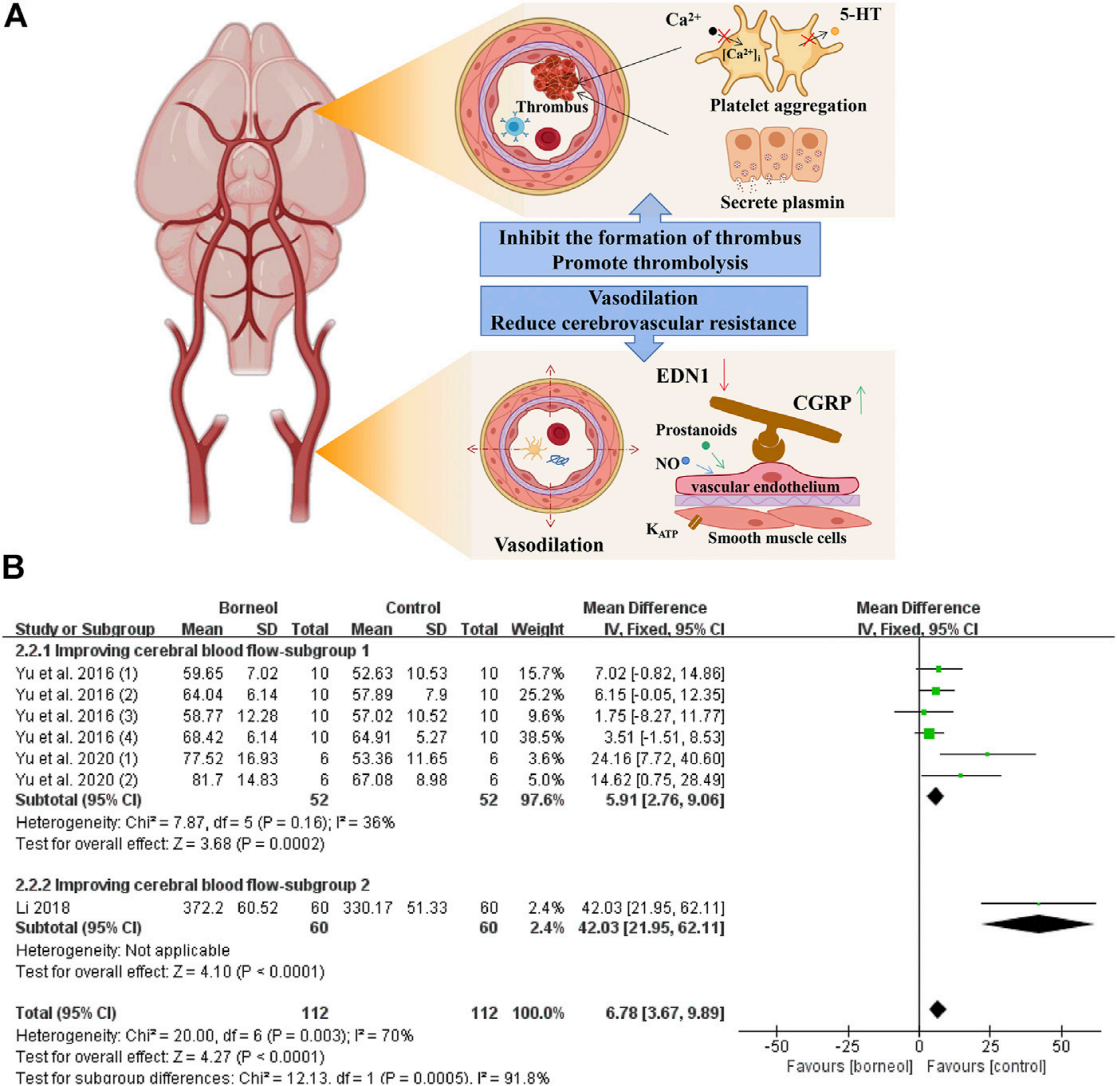
**(A)** Borneol reduces platelet aggregation by blocking the release of 5-HT and the increasing of intracellular Ca^2+^ levels, and interferes with thrombus formation by inhibiting thrombin activity. In addition, borneol activates the fibrinolytic system to promote the dissolution of plasma euglobulin. Borneol also improve cerebral blood flow under ischemia by regulating vascular neuropeptides. **(B)** The forest plots: the borneol group vs. the control group on cerebral blood flow. Subgroup 1 measures the cerebral blood flow in a certain brain area such as the hypothalamus or hippocampus in a tGCIR rodent model, showing that borneol significant increase of cerebral blood flow in the treatment of cerebral ischemic injury (n_T_/n_C_ = 52/52, MD 5.91, 95% CI: 2.76∼9.06, *p* = 0.0002; heterogeneity χ^2^ = 7.87, df = 5, *I* ^2^ = 36%). In subgroup 2, the cerebral blood flow of bilateral parietal cortex was monitored, and the model was induced by photochemical method, which also showed that borneol could improve the cerebral blood flow.

In an *in vivo* canine study, DL-borneol reduced abdominal aortic blood pressure, increased common carotid blood flow, and reduced cerebrovascular resistance (Shang et al., 2015). L-borneol has a vasorelaxant effect that depends on the presence of vascular endothelium and the participation of nitric oxide (NO) and prostanoids. In addition, L-borneol directly relaxed vascular smooth muscle, which is dependent on K_ATP_ channels (Santos et al., 2018). Under pathological conditions, D-borneol increased blood flow in the cortex in photochemical-mediated cerebral ischemia model rats and DL-borneol increased blood flow in the cortex and striatum in transient global cerebral ischemia reperfusion (tGCIR) animal models (Yu et al., 2016; Li, 2018; Wang et al., 2018; Yu et al., 2020). The meta-analysis results of three studies jointly suggested that borneol could improve the blood microcirculation in different brain regions (Figure 3).

Endothelin 1 (EDN1/ET) and calcitonin gene-related peptide (CGRP) are neuropeptides that contract and dilate blood vessels, respectively. Cerebral ischemia/reperfusion injury induces increased EDN1 secretion, promotes vascular smooth muscle contraction, and aggravates low perfusion. Conversely, CGRP is a strong neuropeptide vasodilator that serves as an endogenous endothelin antagonist to reverse vasospasms and improve blood circulation. Stroke leads to increased EDN1 and decreased CGRP levels (Yuan et al., 2006; Giannopoulos et al., 2008). Borneol can effectively reduce EDN1 levels and tends to increase CGRP levels (Huang, 2018), suggesting that it can dilate blood vessels and improve cerebral blood flow during cerebral ischemia.

##### Regulation of the Fibrinolytic System and Anti-Thrombosis

The thrombus causing cerebral ischemia is mainly composed of platelets, leukocytes, erythrocytes, and fibrin (Michael et al., 2016). The dissolution time of plasma euglobulin reflects the activity of fibrinolytic enzymes. Borneol significantly shortens the euglobulin lysis time in rats, reduces the weight of the whole blood clot in mice, and inhibits ADP-induced platelet aggregation in rabbits (He, 2005). Furthermore, borneol inhibits venous thrombosis and arteriovenous shunt in a concentration-dependent manner and exerts anticoagulant activity by prolonging prothrombin time and thrombin time (Li et al., 2008). 5-hydroxytryptamine (5-HT), secreted and released by platelets, plays an important role in platelet aggregation and thrombosis. Ca^2+^, as the second messenger, also plays a key role in the process of platelet activation. The deformation, aggregation and release of platelets are accompanied by the increase in the level of intracellular Ca^2+^ (Wöckel et al., 2008). Borneol inhibits FeCl_3_-induced arterial thrombosis in rats, which involves inhibition of platelet 5-HT release and platelet aggregation, as well as the decrease of cytoplasmic Ca^2+^ level in platelet (Yang et al., 2010). The mechanisms of borneol improves cerebral blood flow and the results of meta-analysis of cerebral blood flow are shown in Figure 3.

#### Inhibition of Neuronal Excitotoxicity

The excessive release of glutamate and the drastic disruption of glutamate transporters, that occurs early after the cerebral ischemic is toxic to neurons, mainly through the activation of ionotropic receptors and intracellular calcium overload that trigger detrimental cascades causing excitotoxic neuronal death (Lai et al., 2014). Conversely, Some *in vitro* studies showed that activation of GABA_A_ and GABA_B_ receptors played a neuroprotective role (Costa et al., 2004; Xu et al., 2008). The expression of GABA_A_ receptors after ischemia is decreased in distinct brain regions of rodents subjected to transient MCAO (Huang and Zhao, 2017). And the disruption of GABA-mediated neurotransmission early during reperfusion may also contribute to ongoing neuronal excitability (Mele et al., 2014). Neuroprotection is achieved in the preclinical setting by GABAergic drugs acting through multiple mechanisms, such as GABA receptors agonists or positive modulators. Borneol plays a neuroprotective role by reducing glutamate (Glu) levels, activating GABA_A_ receptor, and then blocking the necrosis and apoptosis of neurons.

**FIGURE 4:**
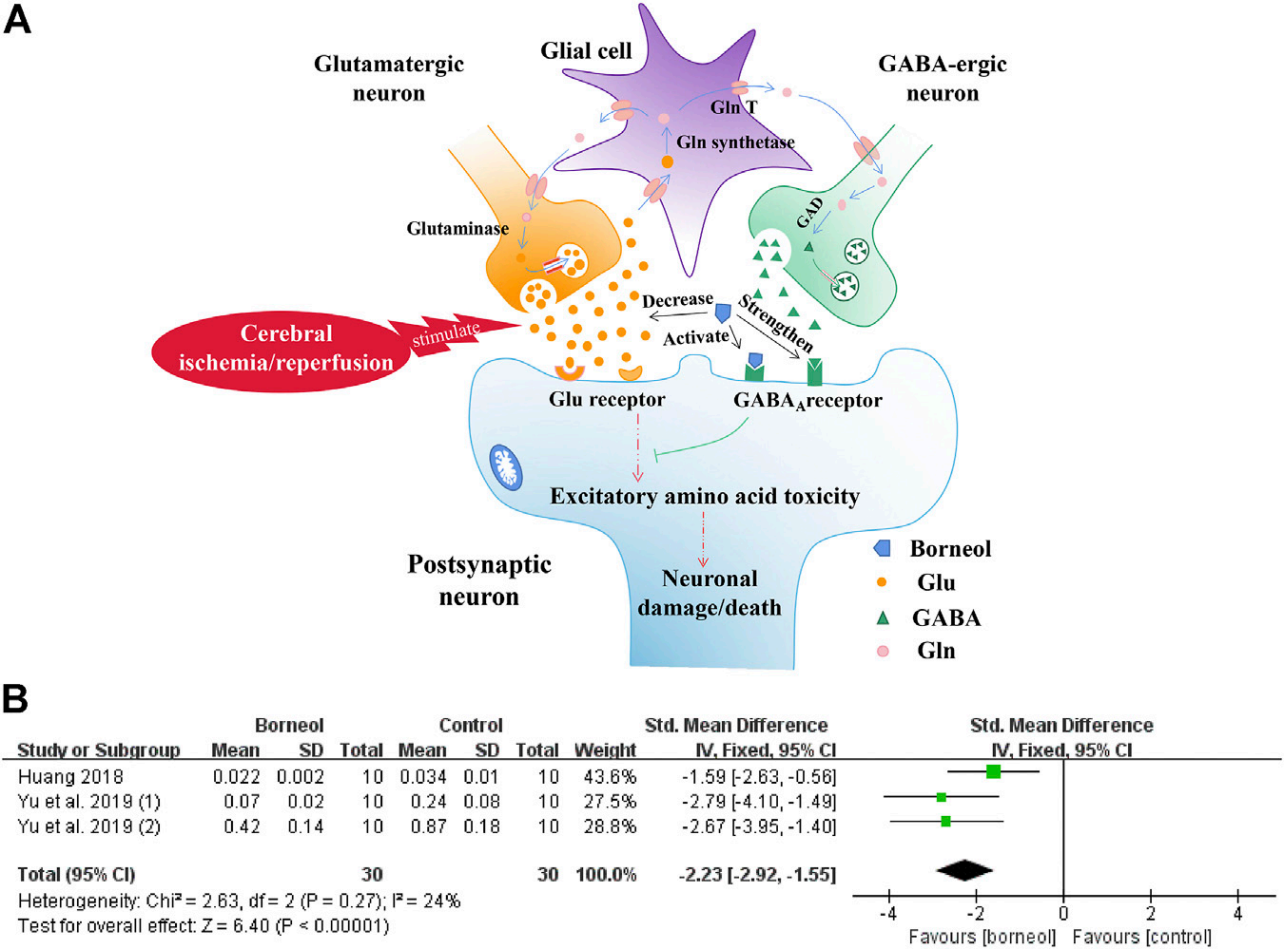
**(A)** Borneol inhibits the increase of extracellular glutamate levels in the ischemic state and strengthens the binding of GABA to GABA_A_ receptors. The direct activation of the GABA_A_ receptor by borneol could also inhibit glutamate-mediated excitatory amino acid toxicity. **(B)** The forest plots: the borneol group vs. the control group on glutamate levels. Meta-analysis of two studies with three comparisons showed that animals in the borneol group had statistically significant lower glutamate levels than the control group (n_T_/n_C_ = 30/30, SMD −2.23, 95% CI: −2.92∼ −1.55, *p* < 0.00001, heterogeneity χ^2^ = 2.63, df = 2, *I* ^2^ = 24%).

In one study, D-borneol and L-borneol enhanced the actions of γ-aminobutyric acid (GABA) at recombinant GABA_A_ receptor and had moderate direct action on these receptors (Granger et al., 2005). D-borneol has neuroprotective effects after primary neuronal injury induced by glutamate at low concentrations, and this effect is consistent with the activation of GABA_A_ receptor (Cheng et al., 2006; Chen et al., 2013). In addition, borneol reduces Glu levels in the hippocampus and hypothalamus in a tGCIR rat model (Yu et al., 2019a). The inhibitory effects of borneol on Glu secretion were also confirmed in a transient bilateral carotid occlusion (tBCO) rodent model (Huang, 2018). The meta-analysis results of the above two studies further suggested that borneol could reduce glutamate levels with small heterogeneity (Figure 4). Borneol promoted glutamate clearance in hypoxia/reoxygenation astrocytes and improved astrocyte viability during hypoxia (Chai et al., 2019). Borneol, combined with echinacoside, artificial moschus, *Ligusticum chuanxiong*, and other drugs, can also improve the content of Glu after cerebral ischemia (Liu et al., 2002a; Zhong et al., 2012; Yu et al., 2019b). The mechanisms by which borneol attenuates the toxicity of excitatory amino acid and the meta-analysis results of glutamate level are shown in Figure 4.

#### Protection Against Damage Caused by Reactive Oxygen Species

The reactive oxygen species (ROS) causing cerebral ischemic injury include superoxide anion (O^2−^), hydroxyl radical (OH^−^), and hydrogen peroxide (H_2_O_2_). Brain tissue is rich in iron ions that catalyze the formation of free radicals, which makes the brain more vulnerable to ROS (González-Ibarra et al., 2011). When thrombolysis exceeds the therapeutic time window, a large amount of ROS is produced by dysfunctional mitochondria, the neutrophil respiratory burst, increased xanthine oxidase formation in capillary endothelial cells, and catecholamine self-oxidation after cerebral ischemia/reperfusion (Huang and Zhao, 2017). Furthermore, cerebral ischemia decreases the ability of the antioxidant system to scavenge ROS. The antioxidant system is composed of glutathione peroxidase (GSH-Px), superoxide dismutase (SOD), catalase (CAT), and other antioxidant enzymes. Improving antioxidant enzymes activity or activating related pathways is an important way to combat ischemic brain injury. Borneol improves the body’s ability to resist ROS by increasing the activity of antioxidant enzymes and activating the nuclear factor erythroid 2-related factor 2 (Nrf2)-antioxidant response element (ARE) signaling pathway.

**FIGURE 5:**
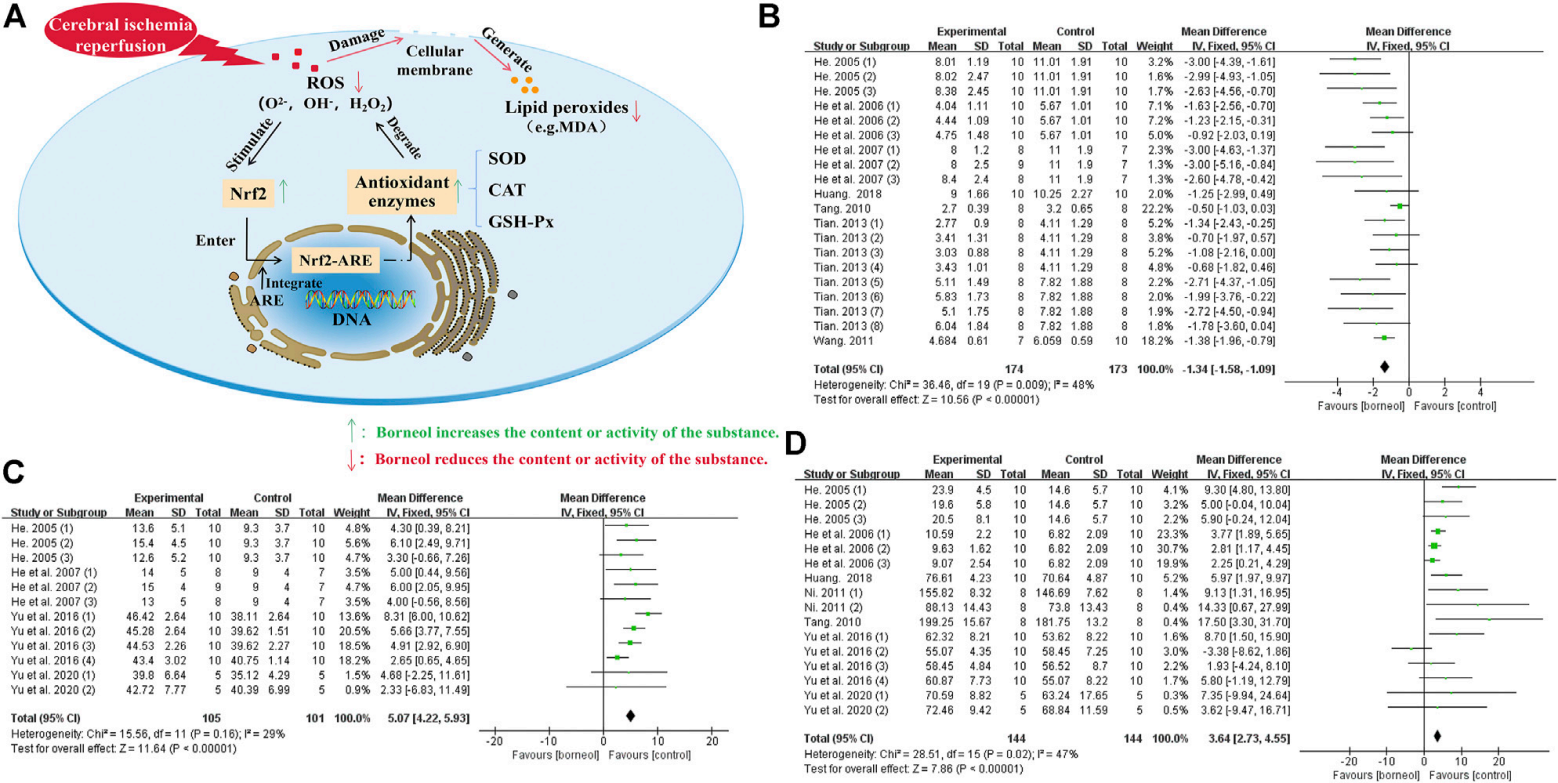
**(A)** Borneol promotes Nrf2 transfer to the nucleus, which activates the Nrf2/ARE signaling pathway by binding to ARE. The activation of this pathway strengthen the expression of downstream antioxidant enzymes such as SOD, CAT, and GSH-Px to reduce the damage of ROS to biofilm and the production of lipid peroxide, maintaining the redox balance of cells. **(B)** The forest plots: the borneol group vs. the control group on MDA levels. After sequentially omitting each study, one outlier study Ni, 2011 was considered as the potential sources of the heterogeneity (n_T_/n_C_ = 190/189, MD −0.86, 95% CI: −1.00 to −0.71, *p* < 0.00001; heterogeneity χ^2^ = 58.37, df = 21, *I* ^2^ = 64%). Meta-analysis of remaining seven studies with 24 comparisons not only showed a more homogeneous result (n_T_/n_C_ = 174/173, MD −1.34, 95% CI: −1.58 to −1.09, *p* < 0.00001; heterogeneity χ^2^ = 36.46, df = 19, *I* ^2^ = 48%), but also displayed a downward effect on the content of MDA. **(C)** The forest plots: the borneol group vs. the control group on GSH-Px activity. The assessments of the GSH-Px activity were performed in four studies with 12 comparisons after excluding one research Huang 2018, heterogeneity before exclusion (n_T_/n_C_ = 115/111, MD 0.20, 95% CI: 0.10 to 0.30, *p* < 0.00001, heterogeneity *χ* ^2^ = 142.43, df = 12, *I* ^2^ = 92%), heterogeneity after exclusion (n_T_/n_C_ = 105/101, MD 5.07, 95% CI: 4.22 to 5.93, *p* < 0.00001, heterogeneity *χ* ^2^ = 15.56, df = 11, *I* ^2^ = 29%). The results showed that animals in the borneol group had statistically significant higher GSH-Px activity than the control group. **(D)** The forest plots: the borneol group vs. the control group on SOD activity. Through 16 comparisons of the 6 studies after excluding the research results of Tian (2013), Shao et al. (2018), and Wang (2011), it was found that borneol had a upward effect on the activity of SOD. Heterogeneity before exclusion (n_T_/n_C_ = 243/248, MD 9.20, 95% CI: 8.51 to 9.89, *p* < 0.00001, heterogeneity *χ* ^2^ = 528.10, df = 27, *I* ^2^ = 95%), and heterogeneity of the 7 retained studies was relatively homogeneous (n_T_/n_C_ = 144/144, MD 3.64, 95% CI: 2.73∼4.55, *p* < 0.00001; heterogeneity χ^2^ = 28.51, df = 15, *I* ^2^ = 47%).

Several studies have shown that borneol injection reduces malondialdehyde (MDA) levels and increases SOD and GSH-Px activity to accelerate the scavenging of superoxide anion free radicals after pMCAO in a rodent model (He et al., 2006; He et al., 2007). Borneol also increases SOD and GSH-Px activity and decreases MDA levels in the cerebral cortex, hippocampus, hypothalamus, and striatum of rats subjected to tGCIR (Yu et al., 2016). L-borneol and DL-borneol increase SOD and decrease MDA in ischemic brain tissue and the serum in a rodent model of tMCAO (Tian, 2013). The meta-analysis results of related studies further suggested that borneol could resist free radical damage by reducing MDA levels and increasing the activities of GSH-PX and SOD (Figure 5). Borneol, combined with musk, *Ligusticum chuanxiong*, and other drugs, can also attenuate ROS injury after cerebral ischemia (Liu et al., 2002b; Wang, 2011; Yu et al., 2017a). Besides, DL-borneol increases SOD and CAT activity, and D-Borneol increases SOD and GSH-Px activity in OGD/R-treated PC12 cells (Huang et al., 2020).

Borneol may protect against free radical damage by activating the Nrf2-ARE signaling pathway. Nrf2, an anti-oxidative stress nuclear transcription factor, translocates from the cytoplasm to the nucleus and binds to the ARE receptor to activate the Nrf2-ARE signaling pathway when cells are exposed to ROS. The activation of this pathway induces the expression of downstream antioxidant enzymes to reduce cell damage caused by ROS and maintain the dynamic cellular redox balance (Wang et al., 2015). Circulating macrophages will enter the core area of cerebral ischemia through the blood-brain barrier to remove damaged cells after organic injury occurs in the brain. An *in vitro* cell experiment demonstrated that D-borneol promotes the expression of Nrf2 in RAW 264.7 macrophages stimulated with lipopolysaccharide (LPS) (Sun et al., 2019). It is also worth mentioning that L-borneol and D-borneol protected human neuroblastoma cells (SH-SY5Y) against β-amyloid induced toxicity, exerted an antioxidative effect by increaseing the expression and nuclear translocation of Nrf2 (Hur et al., 2013). Borneol, combined with *Salvia miltiorrhiza,* not only increased SOD levels and decreased MDA levels but also upregulated the expression of Nrf2 and inhibited the oxidative stress. The combination of these two drugs is more effective than *Salvia miltiorrhiza* alone, suggesting that borneol plays a synergistic role by activating the Nrf2-ARE signaling pathway *in vivo* (Liang et al., 2016). The actions of borneol against ROS damage and the meta-analysis results of related indicators are detailed in Figure 5.

#### Regulation of the Expression of NOS to Protect Against NO Damage

NO plays a dual role in neuroprotection and neurotoxicity. Nitric oxide synthase (NOS) isoforms are crucial in determining the role of NO in cerebral ischemia. There are three isoforms of NOS: neuronal NOS (nNOS/NOS1), inducible NOS (iNOS/NOS2), and endothelial NOS (eNOS/NOS3) (Chen et al., 2017). The transient increase of NO after cerebral ischemia is mainly mediated by eNOS and nNOS. NO synthesized by eNOS can dilate blood vessels, inhibit platelet aggregation, reduce leukocyte adhesion, and enhance collateral circulation to play a short-term neuroprotective effect (Ito et al., 2010). NO synthesized by nNOS participates in glutamate-induced neuronal Ca^2+^ overload, mediates early glutamate excitotoxicity (Zhou and Zhu, 2009). Also, nNOS activation contributes to microvascular damage and decreased cerebral perfusion early after reoxygenation and worsens brain damage (Hsu et al., 2014). The slow upregulation of iNOS in the late stage of cerebral ischemia leads to delayed neuronal injury by producing excessive NO, increasing microvascular permeability, and inducing brain edema (Liu and Mu, 2014). Therefore, promoting the expression of eNOS in the early stage or inhibiting the expression of iNOS in the latter stage is a good therapeutic strategy for treating cerebral ischemia. Studies have found that borneol plays a neuroprotective effect by increasing eNOS expression and reducing iNOS expression, but the effect of borneol on nNOS is not clear.

Intravenous administration of D-borneol inhibits iNOS expression in rodent brain tissue and, consequently, reduces peroxynitrite (ONOO^−^) levels after tMCAO and pMCAO (Wu et al., 2014b; Chang et al., 2017). L-borneol and DL-borneol also reduce the expression level of iNOS in a pMCAO rodent model (Tian, 2013). In an *in vitro* oxygen-glucose deprivation–reperfusion (OGD/R) ischemia model, borneol inhibited the activity and expression of iNOS in primary cortical neurons and, therefore, reduced the production of iNOS-derived NO (Liu et al., 2011). In addition, borneol promotes the synthesis of eNOS-derived NO under both physiological and brain contusion conditions and has a stronger effect on the synthesis of NO by endothelial cells in the pathological state (Zhao et al., 2001). Borneol, combined with catalpol and puerarin, increases the expression of eNOS in rats with MCAO (Wu et al., 2016). The combination of borneol and musk promotes the synthesis of NO in vascular endothelial cells and inhibits the expression of iNOS in a global cerebral ischemia/reperfusion rat model (Zhang et al., 2002; Liu et al., 2011).

#### Inhibition of Intracellular Ca^2+^ Overload

Calcium overload is considered being the last common pathway of neuronal injury during cerebral ischemia/reperfusion (Vannucci et al., 2001; Shi, 2014). The ways of calcium entering neurons following cerebral ischemia include glutamate receptors; voltage-dependent calcium channel; transient receptor potential channels; acid-sensing ion channels; sodium-calcium exchanger operating in entry mode; inward excitotoxic injury current calcium permeable channels; mitochondria and endoplasmic reticulum calcium release, etc. And the pathways of calcium exit into neurons include Ca^2+^-ATPase pump; Na^+^-Ca^2+^ exchanger operating in exit mode, etc (Singh et al., 2019).

**FIGURE 6:**
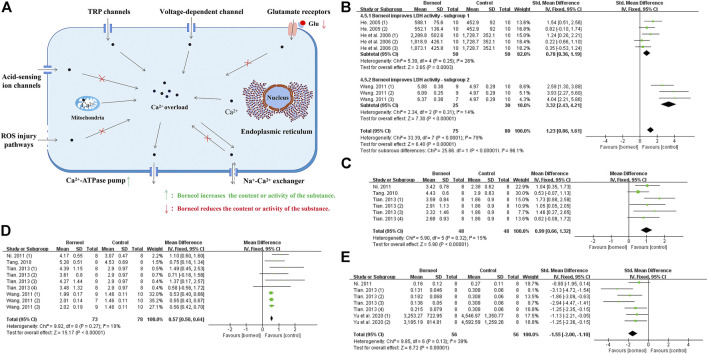
**(A)** Ischemic stroke induces Ca^2+^ overload in the cytoplasm via various mechanisms. Borneol may interfere with calcium overload by interfering with acid ion channels, impeding the damage of ROS to biofilm, reducing glutamate levels, and increasing the activity of Ca^2+^-ATP ase, etc. **(B)** The forest plots: the borneol group vs. the control group on LDH activity. Subgroup analysis was used according to different LDH determination methods, and each subgroup showed that borneol had an improvement effect on LDH activity. **(C)** The forest plots: the borneol group vs. the control group on Ca^2+^-ATP ase activity. A study (He, 2005) were excluded after excluding documents one by one, and the heterogeneity before exclusion (n_T_/n_C_ = 78/78, MD 0.21, 95% CI: 0.13 to 0.30, *p* < 0.00001, heterogeneity *χ* ^2^ = 29.09, df = 8, *I* ^2^ = 72%), after elimination (n_T_/n_C_ = 48/48, MD 0.99, 95% CI: 0.66 to 1.32, *p* < 0.00001, heterogeneity *χ* ^2^ = 5.90, df = 5, *I* ^2^ = 15%). Both meta-analysis results showed that borneol could increase the activity of Ca^2+^-ATP ase. **(D)** The forest plots: the borneol group vs. the control group on Na^+^-K^+^ ATP ase activity. Meta-analysis of four studies with 9 comparisons showed that animals in the borneol group had statistically significant higher Na^+^-K^+^ ATP ase activity than the control group (n_T_/n_C_ = 73/73, MD 0.57, 95% CI: 0.50 to 0.64, *p* < 0.00001, heterogeneity *χ* ^2^ = 9.92, df = 8, *I* ^2^ = 19%). **(E)** The forest plots: the borneol group vs. the control group on Ca^2+^ content. Three studies with seven comparisons indicated that borneol could down regulate the content of Ca^2+^ in brain tissue after cerebral ischemia (n_T_/n_C_ = 56/56, SMD −1.55, 95% CI: 2.00 to −1.10, *p* < 0.00001, heterogeneity *χ* ^2^ = 9.85, df = 6, *I* ^2^ = 39%).

It was found that both L-borneol and DL-borneol significantly reduced Ca^2+^ concentration in ischemic ipsilateral brain tissue of rats subjected to tMCAO (Ni, 2011; Tian, 2013). DL-borneol significantly decreased the concentration of Ca^2+^ in hippocampal and hypothalamic neurons after cerebral ischemia reperfusion (CIR) in rats (Yu et al., 2019a). Previous studies have found that borneol significantly increased the activity of lactate dehydrogenase (LDH) in ischemic brain tissue to inhibit the accumulation of lactic acid, which may interfere with Ca^2+^ entering cells through acid-sensitive ion channels (He, 2005; He et al., 2006; Wang, 2011). Besides, borneol reduces cytoplasmic Na^+^ levels by increasing Na^+^-K^+^ATPase activity, which prevent Ca^2+^ from entering neurons via sodium-calcium exchange (He, 2005). ROS increase cytoplasmic Ca^2+^ concentration by destroying the integrity of the biofilm, but borneol prevents the damage of biofilm by accelerating the scavenging of ROS. Glutamate mediates calcium influx in neurons by activating glutamate receptors, while borneol reduces the level of glutamate (Huang, 2018; Yu et al., 2019b). In addition, L-borneol and DL-borneol increased the expression and activity of Ca^2+^-Mg^2+^-ATPase, promoting the outflow of intracellular Ca^2+^ (Ni, 2011; Tian, 2013). The meta-analysis results also suggested that borneol could reduce the calcium content and increase the activities of LDH, Ca^2+^-ATPase and Na^+^-K^+^-ATPase (Figure 6). Overall, these publications show that borneol can improve the concentration of intracellular Ca^2+^, block the pathological injury caused by Ca^2+^ overload, and interfere with the process of neuronal apoptosis in the cascade reaction. Mechanism of borneol inhibiting cytoplasmic Ca^2+^ overload and the meta-analysis results of related indicators are showed in Figure 6.

#### Suppress Acute Hyperthermia

During the acute stage of cerebral ischemia, fever increases the mortality and disability rates by aggravating neuronal damage (Wrotek et al., 2012). The leukocytes gather in the core area of cerebral infarction and release endogenous heat generators, and these cytokines raise the hypothalamic thermoregulation set point by triggering the release of arachidonic acid and activating cyclooxygenase to produce prostaglandins (Blatteis et al., 2005). Correlation analysis of clinical data and models of cerebral ischemia show that the harmful effects of fever after stroke are mediated by the increased excitotoxicity by glutamate, and the protective effect of hypothermia is also closely related to decreased glutamate release (Campos et al., 2012; Campos et al., 2013). Through feedback regulation, fever can further aggravate cerebral ischemic injury by promoting the secretion of excitatory neurotransmitters, increasing the production of ROS, accelerating the metabolic rate, and aggravating the degradation of neuronal cytoskeleton proteins (Ginsberg and Busto, 1998). Researchers accidentally found that both L-borneol and DL-borneol significantly inhibited the elevation of rats body temperature after pMCAO or tMCAO (Ni, 2011; Tian, 2013). We know that infection can also induce elevated body temperature in stroke patients. Studies have also shown that the three borneols reduced LPS-induced elevation of body temperature and reduced fever due to inflammation (Luo et al., 2016; Zou et al., 2017). These suggest that borneol can inhibit acute fever of ischemic stroke, and this process may involve the inhibition of glutamate excitotoxicity and reduction in the release of endogenous febrile factors.

### Mechanisms of Borneol Intervention in the Subacute Stage of Ischemic Stroke

The physiological and pathological responses in the subacute stage of ischemic stroke (within 2–7°days after the occurrence of human cerebral ischemia) play a key role in the transformation of brain injury in the infarcted area. Apoptosis and necrosis occur in the first few hours after ischemic stroke and reach a peak after 24 h (Wang et al., 2015b). Structural damage to the blood-brain barrier and the high expression of astrocyte aquaporins continue to aggravate brain edema and reach a peak 3–4 days after ischemia (Badaut et al., 2002; Badaut et al., 2007). Compared with the treatment of primary injury after arterial occlusion, secondary injury caused by inflammation may have a longer treatment time window. Intervening in the inflammatory response is an important means of treatment (Kawabori and Yenari, 2015). Therefore, the therapeutic use of borneol for blood-brain barrier damage, different types of cell death, and the inflammatory response in the subacute stage of ischemic stroke have important consequences for the treatment and rehabilitation of stroke patients.

#### Intervention of Inflammatory Cytokine Expression

**FIGURE 7:**
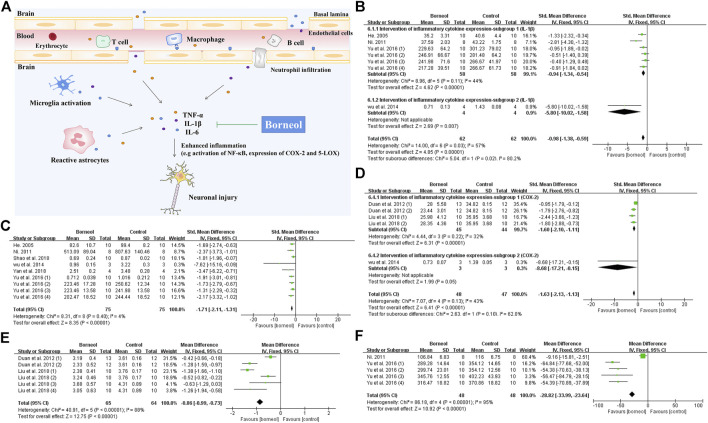
**(A)** Inflammatory cells involved in ischemic brain injury include inherent immune cells in the brain and circulating immune cells infiltrating into the CNS. Borneol attenuates the neuronal damage caused by inflammation via reducing the release of inflammatory cytokines such as TNF-α, IL-1β and IL-6, and down-regulating the expression of COX-2 and 5-LOX, etc. **(B)** The forest plots: the borneol group vs. the control group on IL-1β levels. According to different detection methods, four studies were divided into two subgroups, subgroup 1 heterogeneity (n_T_/n_C_ = 58/58, SMD −0.94, 95% CI: −1.34 to −0.54, *p* < 0.00001, heterogeneity *χ* ^2^ = 8.96, df = 5, *I* ^2^ = 44%) and overall heterogeneity (n_T_/n_C_ = 62/62, SMD −0.98, 95% CI: −1.38 to −0.59, *p* < 0.00001, heterogeneity *χ* ^2^ = 14, df = 6, *I* ^2^ = 57%), suggesting that borneol can reduce the levels of IL-1β levels compared with the control group. **(C)** The forest plots: the borneol group vs. the control group on TNF-α levels. Meta-analysis of six studies showed that animals in the borneol group had statistically significant lower TNF-α levels than the control group (n_T_/n_C_ = 75/75, SMD −1.71, 95% CI: −2.11 to −1.31, *p* < 0.00001, heterogeneity *χ* ^2^ = 8.31, df = 8, *I* ^2^ = 4%). **(D)** The forest plots: the borneol group vs. the control group on expression of COX-2. The expression levels of COX-2 were determined by ELISA and Western bolt analysis in subgroup 1 and subgroup 2, respectively. The results showed that the expression levels of COX-2 in the tested animals was significant decreased after administration of borneol with total heterogeneity (n_T_/n_C_ = 48/47, SMD −1.63, 95% CI: −2.13 to −1.13, *p* < 0.00001, heterogeneity *χ* ^2^ = 7.07, df = 4, *I* ^2^ = 43%). **(E)** The forest plots: the borneol group vs. the control group on expression of 5-LOX. Meta-analysis of two studies with six comparisons showed that animals in the borneol group had significant lower 5-LOX levels than the control group (n_T_/n_C_ = 65/64, MD −0.86, 95% CI: −0.99 ∼ −0.73, *p* < 0.00001, heterogeneity χ^2^ = 40.91, df = 5, *I* ^2^ = 88%). The source of heterogeneity was not found by eliminating the literature one by one. **(F)** The forest plots: the borneol group vs. the control group on IL-6 levels. Meta-analysis of two studies with five comparisons showed that animals in the borneol group had lower IL-6 levels than the control group with substantial heterogeneity (n_T_/n_C_ = 48/48, MD −28.82, 95% CI: −33.99 to −23.64, *p* < 0.00001, heterogeneity *χ* ^2^ = 86.18, df = 4, *I* ^2^ = 95%).

Tumor necrosis factor alpha (TNF-α), interleukin-1β (IL-1β), and interleukin-6 (IL-6) are key inflammatory factors in cerebral ischemic injury. Nuclear factor kappa B (NF-κB) also plays an important role in the inflammatory process (Sun et al., 2014). All kinds of borneol significantly reduce TNF-α levels, while D-borneol and DL-borneol reduce IL-1β levels and DL-borneol reduces IL-6 in cerebral ischemia/reperfusion models (Ni, 2011; Chang et al., 2017; Wen, 2017; Yu et al., 2017b; Dong et al., 2018). Myeloperoxidase (MPO) is a specific marker of neutrophils (Tu et al., 2010). D-borneol inhibits MPO activity, and inhibits neutrophil infiltration in the brain tissue of rats subjected to pBCO (Shao et al., 2018). Inhibition of cyclooxygenase-2 (COX-2) and 5-lipoxygenase (5-LOX) showed a neuroprotective effect in ischemic stroke rat model (Singh and Chopra, 2014). Cerebral ischemia/reperfusion induces the upregulation of COX-2 expression, resulting in the aggravation of the cerebral injury (Zhang et al., 2019). Cysteinyl leukotriene receptor 2 (CYSLTR2) is a subtype of cysteinyl leukotriene receptor, an inflammatory mediator (Zhang, 2013). Borneol inhibits the activities of 5-LOX and COX-2 of rats subjected to tMCAO and blocks CYSLTR2 expression in the hippocampus (Duan et al., 2012; Liu et al., 2018). As shown in Figure 7, the meta-analysis results suggested that borneol played an anti-inflammatory role by reducing the levels of TNF-α, IL-1β and IL-6, and inhibiting the expression of 5-LOX and COX-2 in serum or brain tissue of cerebral ischemic animals. What’s more, borneol inhibits proinflammatory factor release and cytoplasmic inhibitor of kappa B alpha (IκBα) degradation, and blocks NF-κB p65 nuclear translocation induced by OGD/R. Borneol also inhibits the release of TNF-α and the expression of intercellular adhesion molecule-1 (ICAM1), reverses OGD/R-induced neuronal injury, nuclear pyknosis, ROS production, and the disappearance of mitochondrial membrane potential (Liu et al., 2011). Microglia-mediated neuroinflammation plays a crucial role in the pathophysiological process of multiple neurological disorders. Both LPS treatment and oxygen-glucose deprivation of neurons lead to the activation of microglia. Borneol has a direct inhibitory effect on the activation of microglia mediated by LPS *in vitro*. There was an increase in neuronal death after the addition of activated microglia culture supernatant, but the microglia culture medium treated with borneol reduced the toxicity of microglia to neurons with the decrease of TNF-α, IL-1β, and IL-6 levels, and the increase of interleukin-10 (IL-10) levels (Wang et al., 2019). Taken together, these studies described above suggest that borneol protects against acute brain cell injury by attenuating inflammation. The mechanism of borneol attenuating inflammatory injury and the meta-analysis of related indicators are shown in Figure 7.

#### Resistance to Damage of the Blood-Brain Barrier

The blood-brain barrier (BBB) is a selective structural and functional barrier between the brain tissue and blood. The structural barrier is composed of vascular endothelial cells, pericytes, basal lamina, and astroglial terminal feet (Ueno, 2009). The functional barrier depends on P-glycoprotein (P-gp), which can expel specific substances that enter the endothelial cells back into the bloodstream (Ueno et al., 2010). The protective effect of borneol on the BBB is achieved by structurally up-regulating the expression of tight junction proteins in vascular endothelial cells, inhibiting structural destruction of the BBB mediated by metalloproteinases, and functionally inhibiting the efflux of P-gp on neuroprotective drugs.

##### Direct Protective Effect on BBB

**FIGURE 8:**
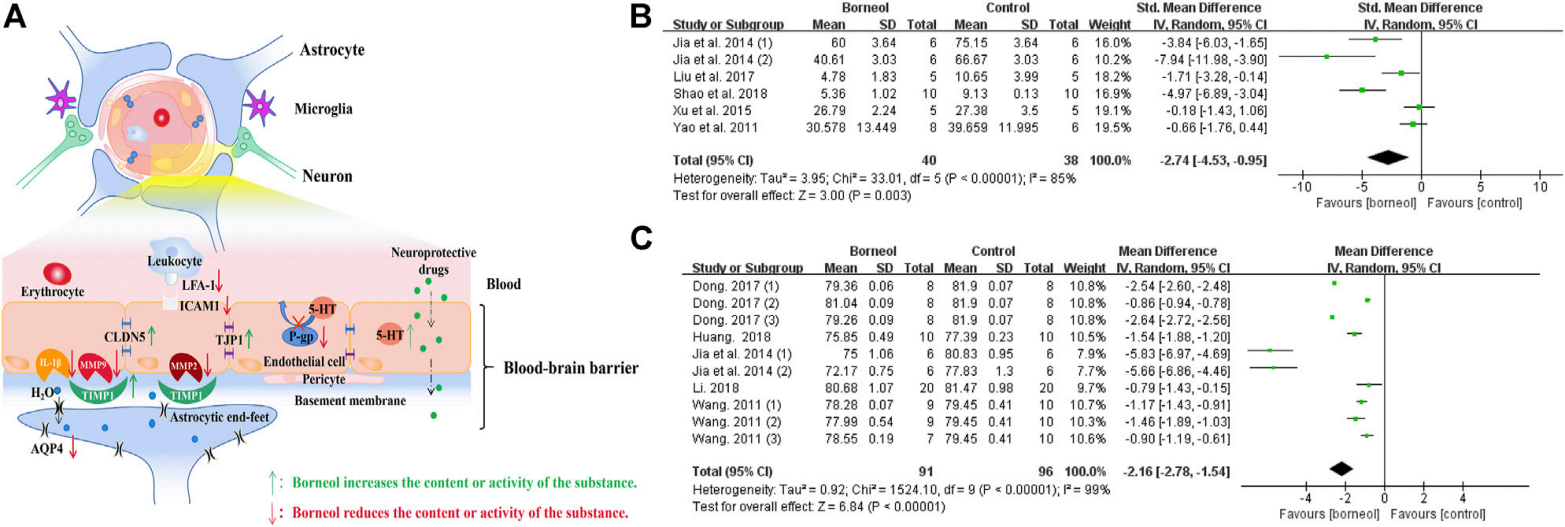
**(A)** Borneol has direct and indirect protective effects on BBB. Borneol inhibits the infiltration of leukocytes to the damaged brain tissue, upregulates the expression of TIMP1 to counter the damage of matrix metalloproteinases to the basement membrane, hinders the destruction of tight junction proteins between endothelial cells, and inhibits the expression of aquaporin. Borneol also plays an indirect neuroprotective effect by inhibiting the efflux of P-gp on neuroprotective drugs and promoting the entry of drugs into the CNS. **(B)** The forest plots: the borneol group vs. the control group on brain EB content. The assessments of the brain EB content were performed in five studies with six comparisons after the induction of the model. Combining available data in a meta-analysis from the above five studies showed the significantly protective effect of borneol on the BBB during cerebral ischemic injury according to the brain EB content (n_T_/n_C_ = 40/38, SMD −2.74, 95% CI: −4.53 to −0.95, *p* < 0.00001, heterogeneity *χ* ^2^ = 33.01, df = 5, *I* ^2^ = 85%). The literature that causes heterogeneity has not been found. **(C)** The forest plots: the borneol group vs. the control group on brain water content. Through 10 comparisons of the five studies assessed brain water content by using dry-wet weight method and showed the significant decreasing of BBB permeability in the treatment of ischemic cerebral injury (n_T_/n_C_ = 91/96, MD −2.16, 95% CI: −2.78 to −0.61, *p* < 0.00001, heterogeneity *χ* ^2^ = 1524.1, df = 9, *I* ^2^ = 99%). One by one exclusion method did not find the heterogeneity from above literature.

The meta-analysis results of several studies confirmed that borneol could reduce the infiltration of evans blue (EB) into the brain and decrease the brain water content after cerebral ischemia (Jia, 2014; Liu et al., 2017; Dong et al., 2018; Wang, 2011; Yao et al., 2011) (Figure 8). The mechanism of borneol inhibits BBB structural damage may involve the downregulation of matrix metallopeptidase 2 (MMP2) and matrix metallopeptidase 9 (MMP9), and the upregulation of TIMP metallopeptidase inhibitor 1 (TIMP1). MMP9 and MMP2 are proteases that promote the opening of the BBB and the formation of brain edema by degrading the basal lamina of cerebral vessels. TIMP1 antagonizes MMP2 and MMP9 activity. DL-borneol increases *TIMP1* mRNA expression in rats subjected to tMCAO (Liu et al., 2007). D-borneol downregulates MMP2 and MMP9 expression in rats subjected to pMCAO, and DL-borneol inhibits *MMP9* mRNA and protein in a tMCAO rat model (Liu et al., 2009; Wang et al., 2018).

In addition to the effects on metalloproteinases, borneol may also downregulate the expression of ICAM1 and lymphocyte function-associated antigen 1 (LFA-1). ICAM1 is a transmembrane protein expressed on vascular endothelial cells that binds to LFA-1 in inflammatory site. The combination of LFA-1 and ICAM1 facilitate leukocyte adherence to, or passage through vascular endothelial cells to reach the focal area of ischemia. Borneol inhibits the adhesion and penetration of leukocytes to endothelial cells by inhibiting the expression of ICAM1 and LFA-1 (Liu et al., 2009; Kong et al., 2013; Liu et al., 2011).

Borneol upregulates claudin 5 (CLDN5) and tight junction protein 1 (TJP1) expression. CLDN5 and TJP1 are key components of tight junctions between cerebral microvascular endothelial cells, and their expression is significantly decreased in ischemic brain tissue (Li et al., 2017). Previous studies have confirmed that D-borneol upregulates CLDN5 and TJP1 expression in tMCAO model rats and brain microvascular endothelial cells with OGD /R treatment (Xu and Zhang, 2015; Xu and Zhang et al., 2016). L-borneol and DL-borneol increased the expression of CLDN5 in an animal model of permanent cerebral ischemia (Xu et al., 2016; Wen, 2017; Dong et al., 2018; Wang et al., 2018).

In addition, aquaporin 4 (AQP4) is concentrated at the foot processes of astrocytes. The outer surface of blood vessels is almost surrounded (85%) by the end feet of glial cells. Thus, increased expression of this protein can induce brain edema (Hu et al., 2012). Borneol inhibits brain edema after cerebral ischemia, which may be related to the downregulation of *AQP4* mRNA expression (Liu et al., 2009).

##### Indirect Protective Effect on BBB

The anatomy of the BBB protects the central nervous system (CNS) from toxins and variations in blood composition, and maintains the consistency of the brain’s micro-environment (Abbott and Friedman, 2012). Although considerable advancements have been made in drug delivery to the CNS, the clinical application of CNS drugs is still limited by their poor bioavailability due to the BBB (Denora et al., 2009). Borneol promotes the penetration and accumulation of neuroprotective drugs such as gastrodin, puerarin, kaempferol, and nimodipine, increases the bioavailability of these drugs, thereby exerting an indirect neuroprotective effect (Cai et al., 2008; Gao et al., 2010; Wu et al., 2014a; Zhang et al., 2015). The mechanism of this effect is closely related to the regulation of P-gp.

One study showed that P-gp was expressed 30 min after focal cerebral ischemia in rats and lasted for 24 h (Xing et al., 2007). Borneol downregulates the expression of P-gp both physiological and pathological conditions. L-borneol inhibits P-gp expression in rats subjected to tMCAO (Yu et al., 2011; Tian, 2013). P-gp, which is encoded by *Mdr1a*, is expressed on the luminal membrane of brain microvascular endothelial cell (BMEC) and confers multidrug resistance to various chemotherapeutic agents (Chen et al., 2003). In an *in vitro* BBB model composed of rat brain BMECs and astrocytes, L-borneol downregulated the efflux function of P-gp by inhibiting the expression of *mdr1a* mRNA and P-gp, and this process was related to the transient activation of NF-κB (Fan et al., 2015).

Borneol also affects 5-HT, a known BBB neurotransmitter (Ke et al., 2000). Borneol can mediate BBB opening by increasing 5-HT in the hypothalamus of rats (Hui et al., 2009). Since P-gp is a lipophilic protein (Abbott et al., 2010), borneol easily binds to P-gp after passing through the BBB and inhibits the binding of 5-HT to P-gp, which results in a reduction in P-gp-mediated efflux of 5-HT and a consequent increase in 5-HT in cerebral vascular endothelial cells (Yuan et al., 2006). The mechanism of borneol protecting BBB and results of meta-analysis of related indicators are shown in Figure 8.

#### Regulation of Various Forms of Cell Death

**FIGURE 9:**
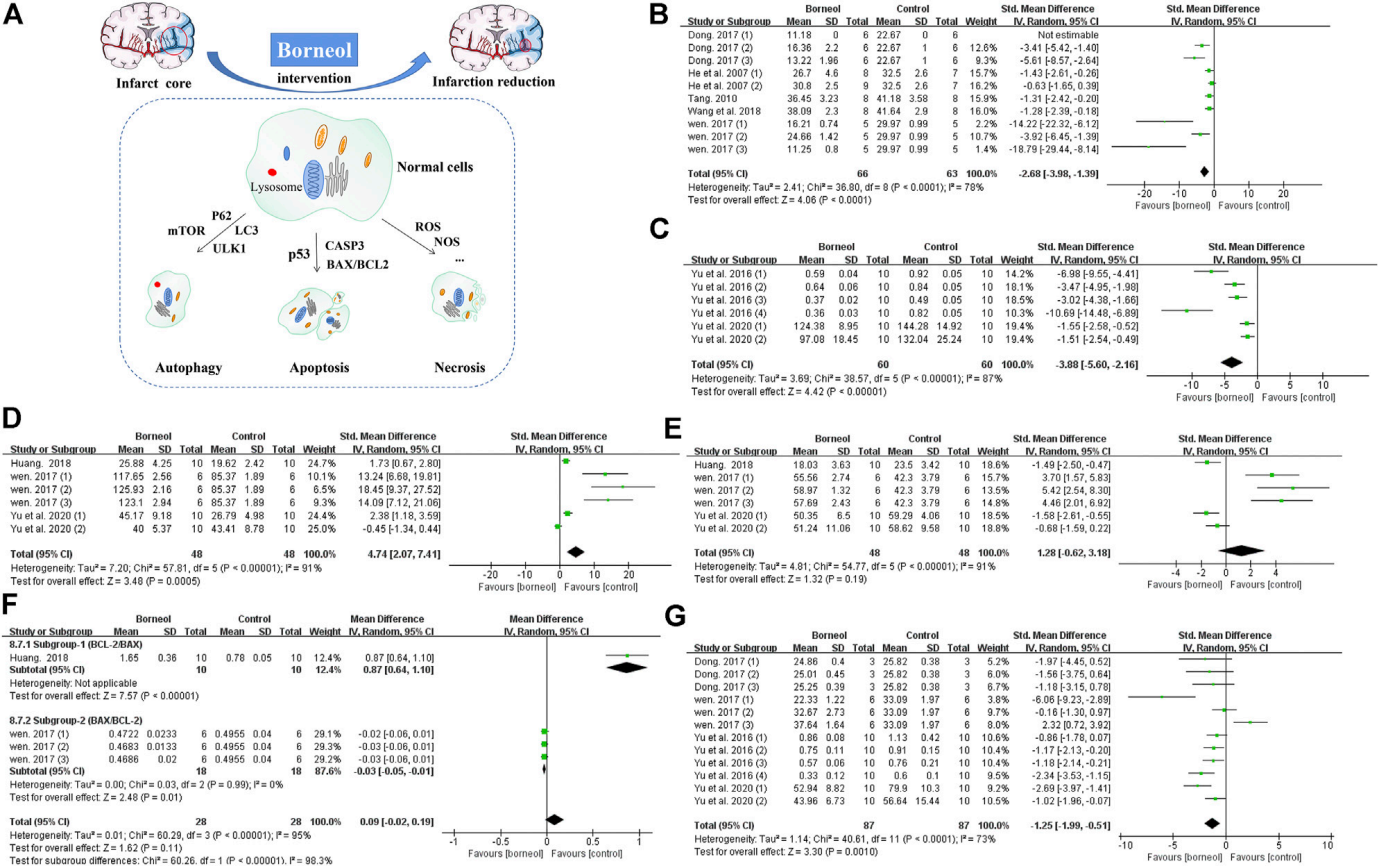
**(A)** Borneol reduces the size of cerebral infarction by improving various forms of cell death. **(B)** The forest plots: the borneol group vs. the control group on cerebral infarction rate. Five studies with ten comparisons to evaluate cerebral infarction size by TTC staining, showing a significant difference but with high heterogeneity (n_T_/n_C_ = 66/63, SMD −2.68, 95% CI: −3.98 to −1.39, *p* < 0.0001, heterogeneity *χ* ^2^ = 36.80, df = 8, *I* ^2^ = 78%). **(C)** The forest plots: the borneol group vs. the control group on expression of *p*53. Meta-analysis of two studies showed that animals in the borneol group had significant lower *p*53 levels than the control group with high heterogeneity (n_T_/n_C_ = 60/60, SMD −3.88, 95% CI: −5.60 to −2.16, *p* < 0.00001, heterogeneity *χ* ^2^ = 38.57, df = 5, *I* ^2^ = 87%). **(D)** The forest plots: the borneol group vs. the control group on expression of BCL2. Three studies showed that animals in the borneol group had statistically significant higher BCL2 levels than the control group with high heterogeneity (n_T_/n_C_ = 48/48, SMD 4.74, 95% CI: 2.07 to 7.41, *p* = 0.0005, heterogeneity *χ* ^2^ = 57.81, df = 5, *I* ^2^ = 91%). **(E)** The forest plots: the borneol group vs. the control group on expression of BAX. Three studies based on the measurement of BAX expression showed no significant difference between the borneol group and the control group with high heterogeneity (n_T_/n_C_ = 48/48, SMD 1.28, 95% CI: −0.62 to 3.18, *p* = 0.19, heterogeneity *χ* ^2^ = 54.77, df = 5, *I* ^2^ = 91%). **(F)** The forest plots: the borneol group vs. the control group on ratio between BCL2 and BAX. Subgroup 1 (n_T_/n_C_ = 10/10, MD 0.87, 95% CI: 0.64–1.10) and subgroup 2 (n_T_/n_C_ = 18/18, MD −0.03, 95% CI: −0.05 to −0.01, *p* = 0.01, heterogeneity *χ* ^2^ = 0.03, df = 2, *I* ^2^ = 0%) are BCL2/BAX and BAX/BCL2, respectively. Both subgroups suggest that borneol can improve the ratio between BAX and BCL2. **(G)** The forest plots: the borneol group vs. the control group on expression of Caspase-3. Expression of Caspase-3 was reported in four studies with 12 comparisons. Meta-analysis showed a significant difference in down-regulating the expression levels of CASP3 but with substantial heterogeneity (n_T_/n_C_ = 87/87, SMD −1.25, 95% CI: −1.99 to −0.51, heterogeneity *χ* ^2^ = 40.61, df = 11, *I* ^2^ = 73%). The above indicators with high heterogeneity have not found their respective resources.

Previous studies applied TTC staining to evaluate cerebral infarction area, showing that borneol had significant differences in alleviating cerebral infarction (Wen, 2017; Dong et al., 2018). The cytotoxic effects of excitatory amino acid toxicity, oxidative stress, and ROS damage in the acute stage of cerebral ischemia lead to rapid disintegration and necrosis of neurons. Delayed neuronal injury after several days of ischemia exhibits characteristics of apoptosis. Cellular injury in the central necrotic area of the cerebral infarction is irreversible, but the peripheral ischemic penumbra is salvageable (Guo, 2000). BCL2 apoptosis regulator (BCL2) and BCL2 associated X, apoptosis regulator (BAX) are typical BCL-2 family apoptosis-inhibiting protein and apoptosis-inducing protein, respectively. Regulating the balance between BAX and BCL2 can hinder the expansion of infarct area (Xu et al., 2016). Previous reports demonstrated that D-borneol and L-borneol reduced the BAX/BCL2 ratio in rats subjected to pMCAO and DL-borneol reduced the BAX/BCL-2 ratio in both pMCAO and tMCAO models (Huang, 2018; Wen, 2017). Caspase-3 (CASP3) induces neuronal apoptosis in ischemic stroke, and which is interrupted by L-borneol and D-borneol after cerebral ischemia/reperfusion (Wen, 2017; Yu et al., 2020). Tumor protein 53 (p53) is mainly expressed in the mitochondria and contributes to cell apoptosis (Wang et al., 2017b). Borneol reduces p53 expression in the cortex and striatum (Yu et al., 2020). Meta-analysis results also suggested that borneol had anti-apoptotic effects by down-regulating the expression levels of p53 and caspase-3, and regulating the ratio of BAX and BCL-2 (Figure 9). Furthermore, borneol enhances the neuron protective autophagy in the cortex and striatum by modulating beclin1, mTOR, and LC3II/I (Yu et al., 2017c; Yu et al., 2020). L-camphor is one of the metabolic products of borneol in the body (Jiang et al., 2008). Several studies have demonstrated that L-camphor upregulates cell adhesion molecule 2 (CADM2) expression to promote neurite outgrowth by targeting microRNA-125a and microRNA-140 and upregulates heterogeneous nuclear ribonucleoprotein A1 (HNRNPA1) expression to promote the expression of stress granules. At the same time, L-camphor can improve apoptosis and autophagy by regulating the autophagy-related proteins, p62 and LC3, and apoptosis-related proteins BCL-XL and CASP3 (Li, 2016; Liu, 2017; Ren, 2018). The mechanism of borneol intervention in various forms of neuronal death and related meta-analysis results are shown in Figure 9.

### Mechanism of Borneol Intervention in the Late Stage of Ischemic Stroke

In the late stage of ischemic stroke (one week after the occurrence of cerebral ischemia in humans), reactive glial cell hyperplasia and glial scar formation limit the expansion of brain damage and separate the necrotic infarct core from the surrounding normal tissues (Sims and Yew, 2017). The reconstruction of the glial cell groups, the neovascularization of the blood vessels, and the regeneration of the nerve myelin sheath play leading roles in the repair process at this stage. Borneol helps the body recover to a healthy physiological state more quickly by promoting angiogenesis and accelerating the repair of damaged neurons in the late stage of stroke.

#### Promoting the Growth and Repair of Neurons and Angiogenesis

Animal experiments confirmed that borneol improved the neurological function score and reduced the cerebral infarction area (Wu et al., 2014b; Dong et al., 2018). Immunofluorescence staining showed that borneol also lowered neuronal mortality and promoted neurogenesis (Zhang et al., 2017b; Yu et al., 2020). Tanshinol borneol ester (DBZ) is a novel synthetic compound derived from Dantonic®. DBZ plays a role in inducing angiogenesis in both *in vivo* and *in vitro* experiments (Liao et al., 2019). Borneol promotes neurogenesis and angiogenesis in the brain, possibly through the upregulation of endogenous neurotrophic factors and regulation of the Wnt/β-catenin pathway.

##### Increasing Endogenous Neurotrophic Factors

Ischemic brain injury destroys nerves and cerebral vessels and induces compensatory neonatal reactions in brain tissue, including nerve regeneration and vascular remodeling. Borneol regulates brain derived neurotrophic factor (BDNF), nerve growth factor (NGF), vascular endothelial growth factor (VEGF), fibroblast growth factor 2 (FGF2/bFGF), and glial cell derived neurotrophic factor (GDNF), and promotes repair of nerves as well as remodeling of blood vessels by activating the Wnt/β-catenin pathway.

In a tMCAO rodent model, DL-borneol significantly decreased *VEGF* mRNA on the first day after cerebral ischemia but increased *VEGF* mRNA levels on the second and third days (Hu et al., 2005; Ni, 2011). Three kinds of borneol can upregulate the expression of VEGF in a pMCAO rat model after ischemia (Dong et al., 2018). Under physiological conditions, endothelial cells express approximately 10 times less fms related receptor tyrosine kinase 1 (FLT1/VEGFR1) than kinase insert domain receptor (KDR/VEGFR2) (DeVal and Black, 2009). Binding of VEGF to VEGFR2 activates intracellular tyrosine kinases and multiple downstream signals that induce angiogenesis. The inhibition of VEGFR2 signaling disturbs endothelial cell proliferation after stroke (Shimotake et al., 2010). On the contrary, VEGFR1 negatively regulates cell proliferation and reduces angiogenesis (Mendes et al., 2018). Increasing evidence suggests that cerebral ischemia upregulates VEGFR1 expression, which positively correlates with the degree of damage (Yoo et al., 2010; Causey et al., 2012). In a BMECs model treated with OGD, borneol increased the expression of VEGF, reduced the expression of VEGFR1, and tended to increase the expression of VEGFR2 (Yu et al., 2019a). The latest research confirms that L-borneol can promote angiogenesis coupled neurogenesis by regulating Ang1-VEGF-BDNF to play a neuroprotective effect (Ma et al., 2021). In a tMCAO rat model, borneol significantly enhanced the expression of *NGF*, *BDNF*, *GDNF*, and *VEGF* mRNA after 48°h of reperfusion. Borneol not only increased the expression of *NGF*, *GDNF*, and *VEGF* mRNA but also significantly increased the level of *bFGF* mRNA after 72 h of reperfusion (Hu, 2004).

##### Regulation of the Wnt/β-Catenin Pathway

The Wnt/β-catenin pathway regulates neuronal differentiation and microangiogenesis, and this pathway is crucial for endothelial cell maintenance of the BBB homeostasis and normal neural function. The Wnt/β-catenin pathway participates in the proliferation and differentiation of neural stem cells, the formation of axons, the occurrence of cortical patterns, vascular regeneration and remodeling in the nervous system, and the formation of the BBB. Wnt family member 3A (WNT3A), β-catenin, disheveled (Dsh), and lymphoid enhancer binding factor 1 (LEF1) are positive regulatory molecules of the Wnt/β-catenin pathway, while glycogen synthase kinase 3 beta (GSK3B) and APC regulator of WNT signaling pathway (APC) are negative regulators of this pathway (Wen et al., 2016). Three kinds of borneol activate the Wnt/β-catenin signal pathway by regulating the expression of positive and negative regulatory molecules in rats subjected to pMCAO, performing the neuroprotective effect of CNS (Wen, 2017). In another study, the combination of borneol, astragaloside IV, and *Panax notoginseng* saponins promotes the proliferation of neurons and repairs damaged neurons, enhancing the resistance of rats to cerebral ischemia/reperfusion injury, which is also associated with the regulation of the Wnt/β-catenin signaling pathway (Yang et al., 2019).

## Discussion

### Summary of Results

Ischemic brain injury is an extremely complex process, but the neuroprotective effect of many drugs is more likely to play a single role in the ischemic cascade reaction. In summary, borneol has protective effects at all three stages of cerebral ischemic injury. In the acute stage of cerebral ischemia, borneol improves cerebral blood flow by relaxing blood vessels and eliciting anti-thrombotic effects, inhibits neuronal excitotoxicity by reducing Glu levels and activating GABA_A_ receptors, improves the ability of the body to resist ROS injury by increasing the activity of antioxidant enzymes, regulates the activity of different types of NOS and antagonizes the neurotoxic effect of NO, reduces the content of intracellular Ca^2+^, interferes with the process of neuron death, and antagonizes the symptoms of acutely elevated body temperature. In the subacute phase of ischemia stroke, borneol interferes with the expression of inflammatory cytokines, the destruction of BBB, and multiple forms of neuron death. In the late stage of brain injury, borneol promotes neuronal repair and angiogenesis. Furthermore, borneol mobilizes endogenous nutritional factors to hasten self-repair of the body, avoiding the defect of single-target therapeutic agents. Moreover, borneol can promote other drugs to pass through the BBB to enhance their therapeutic effects and play a synergistic neuroprotective effect. Hence, brneol can interfere with neuronal injury after cerebral ischemia through multiple channels.

### Implications

Borneol is a naturally occurring product in a class of “orifice-opening” agents used in TCM for resuscitative purpose, and is widely used as an upper ushering drug for various brain diseases in many Chinese herbal formulae (Zhang et al., 2017a; Chen et al., 2019). This is consistent with the findings of many scholars that borneol has a beneficial effect on increasing the bioavailability, tissue distribution, and blood concentration of other drugs, and making other drugs transport through BBB easier (Xiao et al., 2007; Cai et al., 2008; Lu et al., 2011).

Heat-clearing is a traditional effect of borneol as a resuscitation-inducing aromatic medicine (Zou et al., 2017; Wang and Wang, 2018). Some researchers found that oral administration of borneol had a certain improvement effect on fever in the acute phase of stroke, which is consistent with the understanding of “inducing resuscitation with drugs of pungent flavor and cool naturenature” of borneol in TCM clinical practice. But the time-effect, dose-effect relationships, and mechanisms of borneol inhibiting stroke hyperpyrexia deserve a more thorough study.

Oral administration is the most common route in the clinical use of borneol. Borneol is absorbed rapidly into the brain and has the same concentration in the brain as in the blood within 5 min of oral administration (Pan et al., 2014). In mice, a single oral dose of borneol accumulates in organs in this order: lung < muscle < spleen < heart < kidney < brain < liver (Huang et al., 2009). The distribution of borneol in the brain also shows regional specificity, with the highest concentration in the cortex, moderate concentrations in the hippocampus and hypothalamus, and lowest concentration in the striatum (Yu et al., 2013). Besides, the biphasic half-life and elimination of D-borneol and DL-borneol in the plasma were 0.7–8.5 h and 0.8–8.0 h, respectively, (Cheng et al., 2013). Another study found that 10 h after a single dose of 2 g L-borneol, about 81% of borneol is excreted in the form of glucuronic acid binding in urine (Bhatia et al., 2008). These studies suggest that while borneol is easily absorbed, it does not easily accumulate in the body.

### Outlooks

In Table 1, we found that borneol administration in ischemic stroke was mainly oral, with some instances of intravenous administration being recorded. However, it has been reported that intranasal administration of borneol had rapid absorption into the blood and brain compared with oral administration while having similar bioavailability compared to intravenous administration (Zhao et al., 2012). Therefore, it is necessary to pay more attention to the nasal administration of borneol in the future research of brain diseases.

**TABLE 1:** Summary of borneol intervention for ischemic stroke.

Study	Drug	Dosage and delivery way	Animal	Model	Time of ischemia/reperfusion	Outcome measures	Conclusion/possible mechanisms of neuroprotection
Li (2018)	D-borneol	ip, 10 mg/kg	Kunming mice	Photochemical cerebral ischemia model	24 h	(1) Zea-Longa neurological function score	(1) Improve neurological deficits
						(2) Cerebral blood flow (3) Cerebral infarction rate	(2) Reduce brain edema and cerebral infarction size
						(4) Brain water content (5) Neuronal viability	(3) increase blood perfusion
						(6) Caspase-3	(4) Attenuate neuronal apoptosis by downregulating the expression of Caspase-3
						(7) MMP-9	(5) Protect the BBB by downregulating the expression of MMP-9
Wang et al. (2018)	D-borneol	ig, 30 mg/kg	Male, SD rats	pMCAO 2 h	7 day	(1) Longa neurological function score	(1) Reduce cerebral infarction size
						(2) Cerebral blood flow	(2) Increase cerebral blood flow
						(3) Cerebral infarction rate	(3) Improve the brain histopathological morphology
						(4) Cerebral histopathology with H&E staining	(4) Protect the BBB by downregulating the expression of MMP-2,MMP-9 and *MMP-9* mRNA and increasing the expression of ZO-1 and *Claudin-5* mRNA.
						(5) MMP-2	
						(6) MMP-9 and MMP-9 mRNA (7) Claudin-5 mRNA	
						(8) ZO-1	
Yu et al. (2020)	DL-borneol	ig, 80 mg/kg	SD rats (sex in half)	GCIR 60s	7 day	(1) Cerebral blood flow	(1) Improve microcirculation
						(2) SOD, CAT, GSH-Px, MDA, ROS, iNOS and NO	(2) Promote autophagy by increasing the expression of LC3 I/II and Beclin1
						(3) Apoptosis-related genes: p53, Caspase-3, Bcl-2, bax	(3) Inhibite apoptosis in cortex by regulating the expression of p53, Caspase-3, Bcl-2 and bax
						(4) Apoptosis rate with TUNEL staining	(4) Inhibite Ca^2+^ overload
						(5) Autophagy-related proteins:pAMPK, mTOR, ULK1, LC3 I/II, Beclin1 and BNIP3	
						(6) Ca^2+^ content	
Yu et al. (2016)	DL-borneol	ig, 160 g/kg	Male, SD rats	tGCIR 10min	7 day	(1) Cerebral blood flow	(1) Improve microcirculation
						(2) Inflammation indicators: IL-1β, IL-6, TNF-α	(2) Inhibit inflammatory response by decreasing the expression of IL-1β, IL-6, TNF-α
						(3) Antioxidant ability: SOD,GSH-PX, and MDA	(3) Antioxidative damage by increasing the activity of SOD and GSH-PX, and decreasing the MDA content (4) Antiapoptosis by decreasing the expression of p53 and Caspase-3
						(4) Apoptosis-related genes: p53, Caspase-3	
						(5) Score of nissl staining	
Yu et al. (2019b)	DL-borneol	ig, 160 mg/kg	Male, SD rats	tGCIR 20min	7 day	(1) Ca^2+^ content	(1) Reduce glutamate levels and Ca2+ content in hippocampus and hypothalamus
						(2) The ultrastructure of BBB (3) Apoptosis rate with TUNEL staining	(2) Inhibit nerve cell apoptosis and protect BBB ultrastructure
						(4) Levels of glycine, glutamate, and γ-aminobutyric acid	
He 2005 (1)	DL-borneol	iv, 2.0, 1.0, 0.5 mg/kg	Kunming mice (sex in half)	tMCAO 15 min	22 h	(1) Cerebral infarction rate	(1) Reduce cerebral infarction rate
						(2) Neurological function score (3) Step-down test and avoidance reaction experiment	(2) Improve neurological deficits and memory ability
He 2005 (2)	DL-borneol	iv, 1.4 mg/kg	Male and female, Wistar rats	tMCAO 2 h	22 h	(1) Cerebral infarction rate	Anti-inflammation by decreasing the expression of TNF-α,IL-1β, and ICAM-1
						(2) Neurological function score	
						(3) ICAM-1, IL-1β,TNF-α	
He 2005 (3)	DL-borneol	iv, 2.0, 1.0, 0.5 mg/kg	Kunming mice (sex in half)	tMCAO 30 min	2 h	(1) LDH	Improve the energy metabolism disorder by upregulating the activity of Na^+^-K^+^-ATPase,Ca^2+^-ATPase,Mg^2+^-ATPase, and LDH
						(2) Na^+^-K^+^-ATPase	
						(3) Ca^2+^-ATPase and Mg^2+^-ATPase	
He et al. (2006)	DL-borneol	Iv, 0.35, 0.7, 1.4 mg/kg	Kunming mice (sex in half)	tMCAO 3 h	3 h	(1) Neurological function score	(1) Reduce oxidative reactions by increasing the activity of SOD and decreasing MDA levels
						(2) MDA,SOD,LDH	(2) Improve the energy metabolism disorder by upregulating the activity of LDH
He et al. (2007)	DL-borneol	iv, 2.0, 1.0, 0.5 mg/kg	Kunming mice (sex in half)	pBCO	6 h	(1) Cerebral infarction rate	Reduce oxidative reactions by increasing the activity of GSH-Px and decreasing MDA levels
						(2) GSH-Px, MDA	
Huang (2018)	DL-borneol	ig, 100 mg/kg	SD rats (sex in half)	tMCAO 2 h	24 h	(1) Brain water content	(1) Antiapoptosis by modulating the Bax/Bcl-2 expression
						(2) Bax and Bcl-2	(2) Reduce oxidative reactions by increasing the activity of SOD and GSH-Px, and decreasing the concentration of MDA
						(3) ET and CGRP	(3) Dilate blood vessels by increasing CGRP content and reducing ET content
						(4) SOD, MDA, and GSH-Px	(4) Anti-excitatory amino acid neurotoxicity by decreasing levels of glutamate and aspartic acid
						(5) Levels of glycine, glutamate, aspartic acid, and γ-aminobutyric acid	
Tian (2013)	L-borneol	ig, 133.3, 200 mg/kg	Male, SD rats	tMCAO 2 h	22 h	(1) Rate of cerebral edema	(1) Improve the energy metabolism disorder by upregulating the activity of Na^+^-K^+^-ATPase, Ca^2+^-Mg^2+^-ATPase, and T-ATPase
Tian (2013)	DL-borneol	ig, 133.3, 200 mg/kg	Male, SD rats	tMCAO 2 h	22 h	(2) Rectal temperature	(2) Alleviate the pathological BBB disruption by alleviating the damage of the BBB tight junction integrity
						(3) MDA, SOD	(3) Reduce oxidative reactions by increasing the activity of SOD and decreasing MDA levels
						(4) P-GP, MDR1 mRNA	(4) Neuroprotection via P-GP signaling pathway
						(5) The ultrastructure of BBB (6) NO, iNOS, tNOS	(5) Neuroprotection via NO signaling pathway
						(7) T-ATPase,Na^+^-K^+^-ATPase,Ca^2+^-Mg^2+^-ATPase, Ca^2+^ levels	
Ni (2011)	DL-borneol	ig, 200 mg/kg	Male, SD rats	tMCAO 2 h	22 h	(1) The ultrastructure of BBB	(1) Alleviate the pathological BBB disruption by downregulating VEGF and MMP-9
						(2) SOD, MDA	(2) Reduce oxidative reactions by increasing the activity of SOD and decreasing MDA levels
						(3) IL-1β,IL-6, TNF-α	(3) Anti-inflammation by decreasing the expression of TNF-α,IL-1β, and IL-6
						(4) NO, NOS, Ca^2+^ levels	(4) Neuroprotection via NO signaling pathway
						(5) Na^+^-K^+^-ATPase Ca^2+^-Mg^2+^ATPase	(5) Improve the energy metabolism disorder by upregulating the activity of Na^+^-K^+^-ATPase and Ca^2+^-Mg^2+^-ATPase
						(6) MMP-9, VEGF	
Tang (2010)	DL-borneol	ig, 200 mg/kg	Male, SD rats	pMCAO	6 h	(1) Cerebral infarction rate	(1) Reduce oxidative reactions by increasing the activity of SOD and decreasing MDA and NO levels
						(2) Zea-longa neurological function score	(2) Improve the energy metabolism disorder by upregulating the activity of Na^+^-K^+^-ATPase and Ca^2+^-Mg^2+^-ATPase
						(3) Cerebral histopathology with HE staining	
						(4) SOD, MDA, NO	
						(5) Na^+^-K^+^-ATPase, Ca^2+^-Mg^2+^-ATPase	
Wen (2017)	D-borneol, L-borneol, DL-borneol	ig, D-borneol (600 mg/kg), L-borneol (200 mg/kg), DL-borneol (200 mg/kg)	Male, SD rats	pMCAO	24 h	(1) Zea-longa neurological function score	(1) Antiapoptosis by modulating the Bax/Bcl-2 and Caspase-3 expression at both the mRNA and protein levels
						(2) Brain water content	(2) Protect nerve vascular unit by activating Wnt/β-catenin signaling pathway
						(3) Cerebral infarction rate	(3) Anti-inflammation by decreasing the expression of TNF-α
						(4) The ultrastructure of BBB	
						(5) Cerebral histopathology with HE staining	
						(6) VEGF, NGF, TNF-α, IL-1β	
						(7) *Bax*, *Bcl-2*,*Caspase-3* mRNA and protein	
						(8) *APC*, *Dsh1*, *LEF1*, *Wnt3a GSK-3β β-catenin* mRNA and protein	
Dong et al. (2018)	D-borneol, L-borneol, DL-borneol	ig, D-borneol (600 mg/kg), L-borneol (200 mg/kg), DL-borneol (200 mg/kg)	Male, SD rats	pMCAO	24 h	(1) Zea-Longa neurological function score	(1) Anti-inflammation by decreasing the expression of TNF-α
						(2) Brain water content	(2) Antiapoptosis by modulating the Bax/Bcl-2 expression at both the mRNA and protein levels
						(3) Cerebral infarction rate	(3) Alleviate the pathological BBB disruption by upregulating tight junction proteins Claudin-5
						(4) Cerebral histopathology with HE staining	(4) Accelerate the proliferation of vascular endothelial cells by initiating angiogenesis
						(5) The ultrastructure of BBB	
						(6) VEGF levels in the serum	
						(7) TNF-α levels in the serum	
						(8) *Bax* and *Bcl-2* mRNA and protein	
						(9) *Claudin-5* mRNA	
Wu et al. (2014b)	D-borneol	iv, 0.3 mg/kg, 0.8 mg/kg	SD rats	tMCAO 2 h	24 h	(1) mNSS neurological function score (2) Brain water (3) Cerebral infarction rate (4) TNF-α,IL-1βand COX-2 (5) iNOS ONOO^−^	(1) Anti-inflammation by decreasing the expression of TNF-α and IL-1β(2) anti-free radical injury by decreasing the expression of iNOS and ONOO^−^
Xu et al. (2016)	D-borneol	ig, 28 mg/kg	SD rats	tMCAO 2 h	24 h	(1) Brain EB content	Reduces BBB permeability by increasing protein and mRNA expression of ZO-1 and Claudin-5
						(2) *ZO-1* mRNA and protein (3) *Claudin-5* mRNA and protein	
Jia (2014)	D-borneol	ig, 200 mg/kg	Wistar rats (sex in half)	tGCIR 20 min	3 day	(1) Brain EB content	Reduces BBB permeability by increasing protein expression of ZO-1
						(2) Brain water content	
						(3) Number of ZO-1 positive cells (4) Expression of ZO-1 protein	
Chang et al. (2017)	D-borneol	iv, 1 mg/kg	Male, SD rats	Photochemical cerebral ischemia model	24 h	(1) Cerebral infarction rate (2) Neurological function score (3) Grid-walking task and cylinder task	(1) Anti-inflammation by decreasing pro-inflammatory molecules such as iNOS and TNF-α
						(4) dendrite spine length and number	(2) Produce long-term beneficial effect on sensorimotor functions by ameliorating degeneration of dendrites
						(5) TNF-α and iNOS	
Shao et al. (2018)	D-borneol	ig, 500 mg/kg	Rats	tMCAO 2 h	24 h	(1) SOD	(1) Anti-inflammation by decreasing the expression of TNF-α and MPO
						(2) MPO aand TNF-α	(2) Reduce oxidative reactions by increasing the activity of SOD
						(3) Brain EB content	
Yan et al. (2018)	DL-borneol	ig, 500 mg/kg	Rats	BCO 0.5 h	24 h	(1) SOD	(1) Reduce oxidative reactions by increasing the activity of SOD
						(2) MPO and TNF-α	(2) Anti-inflammation by decreasing the expression of TNF-α and MPO
						(3) Brain EB content	
Huang et al. (2001)	DL-borneol	ig, 1000 mg/kg	SD rats (sex in half)	tBCO 40 min	1 h	(1) NO	Reduce oxidative reactions decreasing the levels of NO and lipid peroxidation and increasing SOD activity
						(2) lipid peroxidation	
						(3) SOD	
Wang (2011)	DL-borneol	ig, 66.67 mg/kg, 133.34 mg/kg, 200 mg/kg	SD rats (sex in half)	pBCO	3 h	(1) Brain water content	(1) Reduce oxidative reactions byincreasing the activity of SOD and decreasing the concentration of MDA
						(2) SOD and MDA	(2) Improve the energy metabolism disorder by upregulating the activity of Na^+^-K^+^-ATPase and improving the activity of LDH
						(3) LDH and Na^+^-K^+^-ATPase	
Duan et al. (2012)	DL-borneol	ig, 25 g/kg, 50 mg/kg	Male, SD rats	tMCAO 2 h	24 h	(1) Neurological function score (2) COX-2	Anti-inflammation by decreasing the activity of COX-2 and 5-LOX
						(3) 5-LOX	
Liu et al. (2018)	DL-borneol	ig, 25 g/kg, 50 mg/kg	Male, SD rats	pMCAO	1 day, 3 day	(1) Zea-longa Neurological function score	Anti-inflammation by decreasing the activity of COX-2, 5-LOX and CysLT2
						(2) COX-2	
						(3) 5-LOX	
						(4) CysLT2	
Liu et al. (2007)		ig, 3 mg/kg	Male and female, SD rats	tMCAO 2 h	24 h	(1) Brain water content	Rduce cerebral water content and permeability of BBB
						(2) Brain EB content	
Yao et al. (2011)		ig, 66.67 mg/kg	Male and female, Kunming mice	BCO 20 min	20 min	Brain EB content	Reduce the permeability of BBB

tMCAO, temporary middle cerebral artery occlusion; pMCAO, permanent middle cerebral artery occlusion; BCO, bilateral common carotid artery occlusion; GCIR, global cerebral ischemia and reperfusion; Ip, intraperitoneal administration; Ig, intragastric administration; Iv, intravenous administration.

It is undeniable that only few patients receive thrombolysis within 6–8 h in the treatment of cerebral ischemia, which results in most patients developing permanent cerebral ischemia (Chalela et al., 2007). Regrettably, most experimental studies being focused on transient ischemic stroke (Mcbride and Zhang, 2017; Ma et al., 2020). This phenomenon can also be seen in Table 1. Thus, the comparative study between permanent cerebral ischemia model and ischemia-reperfusion model has far-reaching clinical value for the differential medication of borneol in the treatment of stroke.

As we all know, there are pharmacodynamic differences between the compounds which are optically active isomers (Wermuth, 2015). Borneol is a bicyclic compound with three chiral carbon atoms and several optical isomers. L-borneol and D-borneol are a pair of optical isomers, and their neuroprotective effects in ischemic injury may be different. Consequently, the comparison of pharmacodynamic differences of different borneols in the intervention of ischemic stroke should be one of the focuses of later work. Besides, it is worth noting that there are many commercial species or isomers of borneol, and the botanical taxonomic names used by researchers are not the same, which makes it difficult to compare the experimental results. Therefore, it is also necessary to clarify the botanical source, spatial structure and drug purity of borneol in future experimental studies.

### Limitations

The mechanisms of ischemic brain damage in humans and experimental animals cannot be equal because of species differences. This paper is based on the injury mechanism in humans at different periods (acute stage, subacute stage, and late stage) as a clue to sort out the neuroprotective effect of borneol in experimental models. Therefore, whether borneol can exert the same protective mechanism in clinical practice requires further study.

Some meta-analysis results have high heterogeneity due to experimental animals, the types and doses of borneol, animal models, and other factors. Therefore, exploring the reason that causes substantial heterogeneity and conducting more detailed data analysis will be issues that need to be addressed by relevant practitioners later.

The quality scores for the included studies shown in Table 2. The average quality score of *in vivo* animal studies for meta-analysis was 4.6. Twenty-six studies were peer-reviewed publications, among them, nine studies were published master’s thesis or PhD thesis. Twenty-six studies declared the random allocation. Three studies reported the masked conduct of experiments. None of the included studies described the blinded assessments of outcome. Twenty-four studies described the use of anesthetic without significant intrinsic neuroprotective activity. Fifteen studies stated they compliance with animal welfare regulations. Twenty-six studies described the experimental animals and the model preparation process in comparatively detail. According to the above quality assessment results, we call for more high-quality studies to confirm the neuroprotective effect and mechanism of borneol in the future.

**TABLE 2:** Quality assessment of included studies.

Study years	A	B	C	D	E	F	G	Total
Li (2018)	+	+	+		+	+	+	6
Wang et al. (2018)	+	+			+		+	4
Yu et al. (2020)	+	+			+	+	+	5
Yu et al. (2016)	+	+			+	+	+	5
Yu et al. (2019a)	+	+			+		+	4
He (2005)	+	+			+		+	4
He et al. (2006)	+	+			+	+	+	5
He et al. (2007)	+	+			+	+	+	5
Huang (2018)	+	+			+	+	+	5
Tian (2013)	+	+			+	+	+	5
Ni (2011)	+	+			+	+	+	5
Tang (2010)	+	+			+	+	+	5
Wen (2017)	+	+			+	+	+	5
Dong et al. (2018)	+	+			+	+	+	5
Wu et al. (2014)	+	+	+		+	+	+	6
Xu et al. (2016)	+	+			+		+	4
Jia (2014)	+	+			+	+	+	5
Chang et al. (2017)	+	+	+		+	+	+	6
Shao et al. (2018)	+	+			+		+	4
Yan et al. (2018)	+	+			+		+	4
Huang et al. (2001)	+	+					+	3
Wang (2011)	+	+			+	+	+	5
Duan et al. (2012)	+	+			+		+	4
Liu et al. (2018)	+	+					+	3
Liu et al. (2007)	+	+			+		+	4
Yao et al. (2011)	+	+			+		+	4

A, peer-reviewed publication; B, random allocation; C, blinded conduct of the Experiments; D, blinded assessment of outcome; E, use of anesthetic without significant neuroprotection; F, compliance with animal welfare regulations; G, detailed description of animals and models.

## Conclusion

Although borneol is rarely used alone in the clinical treatment of brain diseases, it cannot be ignored that borneol exerts a significant neuroprotective effect even when used alone *in vivo* and *in vitro* studies. The meta-analysis results of various animal experiments further indicated that borneol has an intervention effect in energy metabolism, inflammatory reaction, apoptosis, and necrosis as well as other processes of cerebral ischemic cascade reaction. This possible explanation for the benefits and the success of borneol in preventing enlargement of infarction and improving neurological function score. Overall, the previous review excessively focused on the regulation of borneol on BBB and ignored its overall effect on the treatment of cerebral ischemia. But this review comprehensively highlights the potential application of borneol as a neuroprotective agent against cerebral ischemia. We strongly believe that the research field of borneol anti-ischemic stroke is still promising.

## Glossary

5-HT: 5-hydroxytryptamine
5-LOX: 5-lipoxygenase
APC: adenomatous polyposis coli
BBB: blood-brain barrier
BDNF: brain-derived neurotrophic factor
bFGF/FGF2: fibroblast growth factor 2
BMEC: brain micro-vascular endothelial cell
Cadm2: bell adhesion molecule 2
CAT: Catalase
CGRP: calcitonin gene-related peptide
CIR: cerebral ischemia reperfusion
CNS: central nervous system
COX-2: cyclooxygenase-2
CysLT-2: cysteinyl leukotriene receptor-2
Dsh: disheveled
EB: evans blue
eNOS: endothelial nitric oxide synthase
ET-1: endothelin-1
GABA: gamma-aminobutyric acid
GDNF: glial cell-derived neurotrophic factor
Glu: glutamic acid
GSH-Px: glutathione peroxidase
GSK-3β: glycogen synthase kinase-3 beta
H_2_O_2_: Hydrogen peroxide
HnrnpA1: heterogeneous Nuclear Ribonucleoprotein A1
ICAM-1: intercellular adhesion molecule-1
IL-10: interleukin-10
IL-1β: interleukin-1β
IL-6: interleukin-6
iNOS: inducible nitric oxide synthase
LC3: microtubule-associated protein light chain 3
LEF1: lymphoid Enhancer-Binding Factor 1
LFA-1: lymphocyte function-associated antigen-1
MDA: malondialdehyde
MMP 2: matrix metallopeptidase 2
MMP 9: matrix metallopeptidase 9
MPO: Myeloperoxidase
mTOR: mammalian Target of rapamycin
NF-κB: nuclear factor-κB
NGF: nerve growth factor
NMDA-R: N-methyl-d-aspartate glutamate receptor
nNOS: neuronal nitric oxide synthase
NO: nitric oxide
NOS: nitric oxide synthase
O^2−^: superoxide anion
OGD/R: Oxygen-Glucose Deprivation/Reperfusion
OH^−^: hydroxyl radical
p53: tumor protein 53
p62: sequestosome-1
pBCO: permanent bilateral carotid occlusion
pCIR: permanent cerebral ischemia reperfusion
P-gp: P-glycoprotein
ROS: reactive oxygen species
SOD: superoxide dismutase
tBCO: transient bilateral carotid occlusion
TCM: traditional Chinese Medicine
TTC: 2,3,5-Triphenyltetrazolium chloride
tGCIR: transient Global cerebral ischemia reperfusion
TIMP-1: tissue inhibitor of metalloproteinase-1
tMCAO: transient middle cerebral artery occlusion
TNF-α: necrosis factor-α
VEGF: vascular endothelial growth factor
VEGFR1/FLT1: fms related receptor tyrosine kinase 1
VEGFR2/KDR: Kinase insert domain receptor
Wnt3a: wnt family member 3A
